# Intramolecular Interaction Influences Binding of the Flax L5 and L6 Resistance Proteins to their AvrL567 Ligands

**DOI:** 10.1371/journal.ppat.1003004

**Published:** 2012-11-29

**Authors:** Michael Ravensdale, Maud Bernoux, Thomas Ve, Bostjan Kobe, Peter H. Thrall, Jeffrey G. Ellis, Peter N. Dodds

**Affiliations:** 1 Division of Plant Industry, Commonwealth Scientific and Industrial Research Organization, Canberra, ACT, Australia; 2 School of Chemistry and Molecular Biosciences, Australian Infectious Diseases Research Centre, and Institute for Molecular Bioscience, University of Queensland, Brisbane, Queensland, Australia; Purdue University, United States of America

## Abstract

*L* locus resistance (R) proteins are nucleotide binding (NB-ARC) leucine-rich repeat (LRR) proteins from flax (*Linum usitatissimum*) that provide race-specific resistance to the causal agent of flax rust disease, *Melampsora lini*. L5 and L6 are two alleles of the *L* locus that directly recognize variants of the fungal effector AvrL567. In this study, we have investigated the molecular details of this recognition by site-directed mutagenesis of AvrL567 and construction of chimeric L proteins. Single, double and triple mutations of polymorphic residues in a variety of AvrL567 variants showed additive effects on recognition strength, suggesting that multiple contact points are involved in recognition. Domain-swap experiments between L5 and L6 show that specificity differences are determined by their corresponding LRR regions. Most positively selected amino acid sites occur in the N- and C-terminal LRR units, and polymorphisms in the first seven and last four LRR units contribute to recognition specificity of L5 and L6 respectively. This further confirms that multiple, additive contact points occur between AvrL567 variants and either L5 or L6. However, we also observed that recognition of AvrL567 is affected by co-operative polymorphisms between both adjacent and distant domains of the R protein, including the TIR, ARC and LRR domains, implying that these residues are involved in intramolecular interactions to optimize detection of the pathogen and defense signal activation. We suggest a model where Avr ligand interaction directly competes with intramolecular interactions to cause activation of the R protein.

## Author Summary

The biotrophic fungus *Melampsora lini* is the causal agent of flax rust disease. Flax produces immune-receptor proteins that recognize fungal effector proteins, and subsequently signal the activation of plant defense responses. Here we report the molecular details of interactions between *L*-locus immune-receptors and *AvrL567*-locus effectors, as well as the engineering of an enhanced flax immune-receptor. In order to investigate the role of AvrL567 amino acid residues hypothesized to mediate interactions with *L*-locus immune receptors, we generated a series of site-direct mutations in AvrL567 proteins. Conversely, to investigate the role of regions hypothesized to mediate interactions with AvrL567 effectors, we generated a series of chimeric *L*-locus immune-receptors that contain swaps between, and within protein domains. Interactions between modified immune-receptors and effector proteins were evaluated using the yeast-two-hybrid system and transient expression *in planta*. Our results revealed that interactions between *L*-locus immune receptors and *AvrL567*-locus effector proteins involve multiple surfaces, and that intramolecular interactions between, and within, domains of *L*-locus immune-receptors plays a crucial role in these interactions. Finally, the generation of an enhanced immune-receptor is an important proof-of-concept demonstrating the utility of protein engineering in generating novel disease resistance in agricultural crops.

## Introduction

The plant immune system is based upon the ability to accurately perceive and appropriately respond to potential threats. In general, plants use membrane-spanning proteins with extracellular receptor domains to recognize common features of plant pathogens (pathogen associated molecular patterns, PAMPs) and intracellular receptors to detect pathogen effectors transferred into plant cells during infection [1], [2], [3]. Most intracellular immune receptors (disease resistance proteins) contain nucleotide-binding (NB) and leucine-rich repeat (LRR) domains; one subclass of these has a coiled-coil (CC) domain and the other possesses a TIR (Toll, interleukin-1 receptor, resistance protein) domain at the N-terminus [4], [5].

Plant NB-LRR disease resistance proteins belong to the STAND (signal transduction ATPases with numerous domains) clade of AAA+ (ATPase associated with diverse cellular activities) proteins, and are similar to the Nod-like receptor (NLR) family of proteins that act as intracellular surveillance molecules in animal innate immunity [6], [7], [8]. The signature catalytic core of STAND proteins comprises an αβα NB domain, a four-helix ARC1 (APAF-1, R protein, CED-4) domain, and a winged helical ARC2 domain [9], [10], [11]. This domain is thought to function as a reversible molecular switch during signal transduction, with monomeric ADP-bound forms representing the off - or closed - state, and ATP-bound multimeric forms representing the on - or open – state [9], [11], [12], [13]. Tight regulation of this switch is critical in plant NB-LRRs, because these proteins regulate an apoptotic process. The trigger for the conformational change to the open state is generated by signal perception, either directly when NB-LRRs bind effector proteins [14], [15], [16], [17], [18], [19], or indirectly when NB-LRRs detect the biochemical fingerprint of effector proteins as they attempt to carry out their virulence function [20], [21], [22], [23], [24], [25]. This effector-mediated R protein activation is believed to ultimately lead to conformation changes that expose the N-terminal TIR or CC signalling domains, so they can interact with downstream signalling partner [12], [26].

The C-terminal LRR domain of R proteins generally mediates signal perception [27], [28]. This domain is composed of repeating LRR units that form stacking β-strands, resulting in a horseshoe-shape molecule with a continuous, parallel β-sheet on the inner concave surface [29]. Individual LRR units contain xxLxLxx motifs generating β-strand/β-turn structures in which the variable non-leucine residues form the concave, solvent-exposed surface of the horseshoe and are available for participation in protein-protein interactions [29], [30]. This region of plant R proteins is often highly variable, as a result of diversifying selection, and a number of studies have demonstrated changes in specificity mediated by polymorphisms in the LRR domain [18], [30], [31], [32], [33], [34], [35], [36], [37], [38].

TIR-NB-LRR resistance proteins in flax (*Linum usitatissimum*) confer resistance to the flax rust fungus *Melampsora lini* through recognition of effector proteins delivered into the host cell during infection [39], [40]. For example, the *L* resistance locus consists of a single gene encoding 13 allelic protein variants (L, L1 to L11, and LH) that recognise different matching avirulence proteins [32]. L alleles share greater than 90% amino acid sequence identity, with positively selected variation concentrated in the LRR domain. Domain-swap experiments between the L2, L6 and L10 alleles showed that these recognition specificities are determined by the LRR domain [30], [32]. Similarly, the L6 and L11 proteins differ by only 32 amino acids, all in the LRR domain, and a chimeric protein with 11 amino acid changes in the C-terminal region of the LRR displayed a novel specificity, with a reduced recognition spectrum [16], [41].

The L5, L6 and L7 proteins recognise allelic variants of the *M. lini* effector protein AvrL567, a 127-amino acid secreted protein that is expressed in haustoria and translocated into host cells during infection [42], [43], [44]. Seven of the 12 variant forms of AvrL567 (-A, -B, -D, -E, -F, -J, -L) are avirulence alleles as they induce an *L5* and/or *L6*, and/or *L7*-dependent hypersensitive response (HR) in transient expression assays whereas the other 5 variants (-C, -G, -H, -I, -K) are virulence alleles as they do not induce an HR [16]. Yeast-two-hybrid (Y2H) assays demonstrated that AvrL567 and L5, L6, and L7 interact directly and that the specificity of this protein-protein recognition corresponds with that of the HR-inducing recognition *in planta* [16]. L6 and L7 are differentiated by just 11 polymorphisms found in the TIR domain and have identical AvrL567 recognition specificities, although L7 shows consistently weaker interaction in yeast, and a weaker HR *in planta* [26], [30]. L5 and L6 are two of the most diverged L proteins, differing by 89 amino acid polymorphisms (61 in the LRR) and four small indels, but nevertheless have overlapping recognition specificities. They are distinguished by L6 interacting with AvrL567-D, while L5 does not.

Wang *et al*. [45] determined the structures of AvrL567-A and -D and identified four polymorphic surface-exposed amino acid residues that were important for their differential recognition. Here we have further investigated the role of these surface-exposed amino acids in recognition of AvrL567. Single, double and triple mutations at these sites in a variety of AvrL567 variants showed additive effects on recognition strength, suggesting that multiple contact points are involved in the recognition event. We show by domain-swap experiments that the L5 and L6 specificities are determined by their corresponding LRR regions, with contributions made by seven and four N- and C-terminal LRR units, respectively, of a total of 26, where most positively selected amino acid sites occur. This further confirms that multiple, additive contact points occur between AvrL567 variants and either L5 or L6. However we also observed that recognition of AvrL567 is affected by co-operative polymorphisms between both adjacent and distant domains, including the TIR, ARC and LRR domains, implying that these residues are involved in intramolecular interactions to optimize detection of the pathogen and/or defense signal activation.

## Results

### R-Avr recognition: gain- and loss-of-function mutants of AvrL567

**Figure 1:**
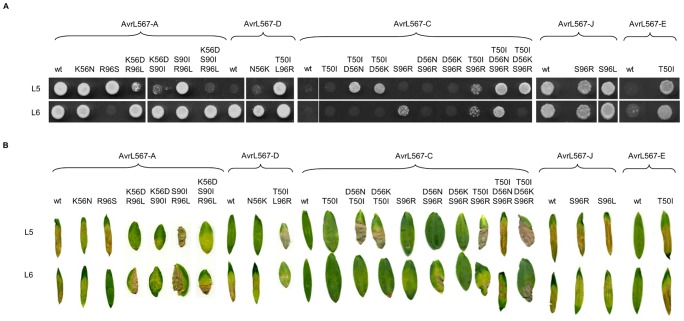
Mutational analysis of AvrL567 interactions with L5 and L6. **A.** Growth of yeast strain HF7c expressing L5 or L6 proteins fused to the GAL4 activation domain and either wild-type (wt) or mutant AvrL567 proteins (amino acid changes specified) fused to the GAL4 DNA-binding domain. Cultures were grown on selective media lacking tryptophan, leucine and histidine and scored after 4 days. **B.** *Agrobacterium-*mediated transient expression of AvrL567-A, -D, -J, -E, -C, and derived mutants, in leaves of near-isogenic flax lines possessing either *L5* or *L6* (cv. Bison). Images were taken 8 d after infiltration.

Sequence comparisons of the 12 AvrL567 variants suggested that polymorphisms at four positions (50, 56, 90 and 96) were associated with specificity differences [16]. Single amino acid substitutions at positions 50 (T50I) or 96 (L96R) were sufficient to restore recognition of AvrL567-D by L5, while the I50T substitution almost completely blocked L5 and L6 recognition of AvrL567-A (Table 1) [45]. To further evaluate the role of these residues in mediating interactions with L5 and L6, we made reciprocal single, double and triple substitutions of these amino acids in a wider range of AvrL567 variants (-A, -D, -E, -J and -C), which show varying recognition patterns (Table 1). Mutant AvrL567 proteins were assayed for recognition by L5 and L6 using both Y2H assays - to test for protein interaction - and by *Agrobacterium-*mediated transient expression *in planta -* to measure *R* gene-dependent cell death (Figure 1 and Table 1).

**Table 1:** The amino acid residues present at positions 50, 56, 90 and 96 in AvrL567 variant proteins is shown along with the L5, L6 and L6RVL11 recognition specificity observed in the yeast-two-hybrid assay.

	Amino acid position				Protein interaction in yeast-2-hybrid		
	50	56	90	96	L5	L6	L6L11RV
***AvrL567-A***	**I**	**K**	**S**	**R**	**+**	**+**	**−**
*AvrL567-A I50T*	**T**	**K**	**S**	**R**	**+/−**	**+/−**	
*AvrL567-A K56D*	**I**	**D**	**S**	**R**	**+**	**+**	
AvrL567-A K56N	**I**	**N**	**S**	**R**	**+**	**+**	
*AvrL567-A S90I*	**I**	**K**	**I**	**R**	**+**	**+**	
*AvrL567-A R96L*	**I**	**K**	**S**	**L**	**+**	**+**	
AvrL567-A R96S	**I**	**K**	**S**	**S**	**+**	**−**	
AvrL567-A K56D;S90I	**I**	**D**	**I**	**R**	**+/−**	**+**	
AvrL567-A K56D;R96L	**I**	**D**	**S**	**L**	**+/−**	**+**	
AvrL567-A S90I;R96L	**I**	**K**	**I**	**L**	**+**	**+**	
AvrL567-A K56D;S90I;R96L	**I**	**D**	**I**	**L**	**−**	**+**	
***AvrL567-D***	**T**	**N**	**I**	**L**	**−**	**+**	**−**
*AvrL567-D T50I*	**I**	**N**	**I**	**L**	**+**	**+**	
*AvrL567-D N56D*	**T**	**D**	**I**	**L**	**−**	**+**	
AvrL567-D N56K	**T**	**K**	**I**	**L**	**−**	**+**	
*AvrL567-D I90S*	**T**	**N**	**S**	**L**	**−**	**+**	
*AvrL567-D L96R*	**T**	**N**	**I**	**R**	**+**	**+**	
*AvrL567-D L96S*	**T**	**N**	**I**	**S**	**−**	**−**	
AvrL567-D T50I;L96R	**I**	**N**	**I**	**R**	**+**	**+**	
***AvrL567-C***	**T**	**D**	**S**	**S**	**−**	**−**	**−**
AvrL567-C T50I	**I**	**D**	**S**	**S**	**−**	**−**	
*AvrL567-C D56N*	**T**	**N**	**S**	**S**	**−**	**−**	
*AvrL567-C D56K*	**T**	**K**	**S**	**S**	**−**	**−**	
AvrL567-C T50I;D56N	**I**	**N**	**S**	**S**	**+**	**−**	
AvrL567-C T50I;D56K	**I**	**K**	**S**	**S**	**+**	**−**	
AvrL567-C S96R	**T**	**D**	**S**	**R**	**−**	**+/−**	
AvrL567-C D56N;S96R	**T**	**N**	**S**	**R**	**−**	**−**	
AvrL567-C D56K;S96R	**T**	**K**	**S**	**R**	**−**	**−**	
AvrL567-C T50I;S96R	**I**	**D**	**S**	**R**	**+/−**	**+/−**	
AvrL567-C T50I;D56N;S96R	**I**	**N**	**S**	**R**	**+**	**+**	
AvrL567-C T50I;D56K;S96R	**I**	**K**	**S**	**R**	**+**	**−**	
**AvrL567-J**	**I**	**N**	**S**	**S**	**+**	**+**	**+**
AvrL567-J S96R	**I**	**N**	**S**	**R**	**+**	**+**	**+**
AvrL567-J S96L	**I**	**N**	**S**	**L**	**+**	**+**	**+**
**AvrL567-E**	**T**	**N**	**S**	**S**	**−**	**+/−**	**−**
AvrL567-E T50I	**I**	**N**	**S**	**S**	**+**	**+**	**+**

Italicized text indicates data from Wang *et al.* [45] or Dodds *et al.* [16].

−indicates no interaction, +indicates an interaction, +/−indicates a weak interaction.

With one exception (see below), single amino acid changes at positions 56, 90 and 96 in AvrL567-A did not alter recognition by L5 or L6 [45]. However, double and triple substitutions at these positions revealed that they all contribute additively to recognition. The K56D/S90I and K56D/R96L double mutants both substantially reduced recognition by L5, while the K56D/S90I/R96L triple substitution blocked L5 recognition completely (Figure 1). Notably, none of these changes affected L6 recognition. Conversely, the single amino acid R96S substitution abolished L6 but not L5 recognition. This indicates that L5 and L6 recognise different molecular features of AvrL567, but at similar positions. For AvrL567-D, Wang *et al.* [45] showed that either T50I or L96R substitutions were sufficient to allow interaction with L5; but we now found that the T50I/L96R double mutation shows an additive effect relative to the single mutants, which can be detected when the GAL4 AD and BD fusions are reversed (Figure S1). Further evidence for additive interactions comes from the context-dependent effects of several single substitutions. For instance, the presence of S or L at position 96 does not prevent L5 recognition of AvrL567-A or -J, but in AvrL567-D an R is required at this position to establish L5 recognition. Likewise, a S96 substitution destabilizes L6 recognition of both -A and -D, but is compatible with L6 recognition of -J (Figure 1 and Table 1).

To complement these loss-of-function studies, we also tested the effect of reciprocal changes in the virulence allele, AvrL567-C, which is not recognised by L5 or L6 and found that multiple amino acid changes were required to restore full recognition (Figure 1, Table 1). For instance, double substitutions at positions 50 and 56 or positions 50 and 96 were required to allow L5 recognition of this protein. L6 recognition could be restored weakly (in yeast but not *in planta*) by the single S96R substitution, but required the triple T50I/D56N/S96R substitution for full recognition. Interestingly, in the context of AvrL567-C, a K residue at position 56 was not compatible with L6 recognition, although it does not prevent recognition in the AvrL567-A or -D contexts (Table 1). The strong positive effect of isoleucine at position 50 was confirmed as the single T50I substitution in AvrL567-E restored its interaction with L5 and L6. These data further support the additive roles of these amino acid positions in recognition.

All AvrL567 mutant fusion proteins were stably expressed in yeast (Figure S2) indicating differential recognition of mutants by L proteins was due to differences in their surface properties, resulting in physical changes in the interactions of these proteins. Data from Y2H and *in planta* HR analyses correlated well, apart from a few exceptions, which can all be explained by *in planta* HR induction being less sensitive than the Y2H interaction (for instance, L6 interactions with AvrL567-A K56N or AvrL567-J S96L; Figure 1). Overall, these data suggest that multiple contact points at disparate positions on the AvrL567 molecule are involved in interaction with the corresponding R proteins and make additive contributions to the strength of recognition. In addition, although L5 and L6 recognition of AvrL567 involves contacts with similar positions, they have different requirements for amino acid residue features at these positions. The cloned *Avr* genes and their derived mutants provide a sensitive set of test proteins to detect subtle changes of specificity of L5–L6 chimeric proteins described in the following sections.

### Positively selected sites are concentrated in the LRR and ARC1 domains of *L* locus proteins

**Figure 2:**
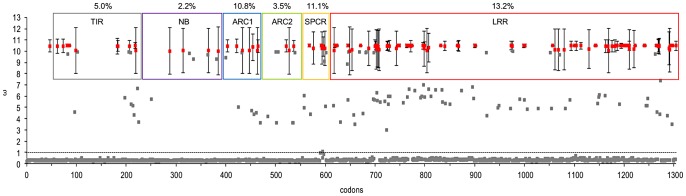
Scatter plot of ω values of codons found in the TIR (grey box), NB (purple box), ARC1 (blue box), ARC2 (green box), spacer (SPCR; orange box) and LRR (red box) regions of *L* locus R proteins. Codons with statistically significant (*p*>0.95) ω values are represented by red dots with error bars representing the standard error of the mean. The percentage of statistically significant positively selected codons is indicated above each region. The dashed line indicates a ω value of 1.

In order to correlate the recognition-determining residues in the AvrL567 proteins with variation in the L5 and L6 proteins, we conducted an analysis of positive selection on the coding sequences of all 12 cloned *L* genes. Previous analysis had found an excess of non-synonymous versus synonymous substitutions in the LRR domain [30], and we used the program CODEML [46] to identify codons under positive selection. The M8 model allowing for positive selection provided a significantly better fit to the data than the M7 null hypothesis model, which excludes positive selection (*p*<0.001; Table S1) and predicted 123 (9.5%) codons as being under significant positive selection (Figure 2). This includes 86 of the 99 sites polymorphic between L5 and L6 (Figure S3). To examine the distribution of positively selected sites we considered the protein sequence in six regions: the TIR, NB, ARC1, ARC2 and LRR domains and a short spacer region between ARC2 and LRR domains. In the LRR domain, 13.2% of codons (92 sites) are under significant positive selection, compared to only 5.3% of codons (31 sites) in the rest of the protein. These occurred mainly in the N-terminal and C-terminal portions of the LRR domain, with a lack of positively selected sites in the central portion of the LRR domain. This suggests that recognition specificity may be conferred mainly by interactions involving the two extremities of the LRR domain. The ARC1 domain and the spacer also showed elevated numbers of positively selected sites (about 11%) compared to the TIR, NB and ARC2 domains (2 to 5%; Figure 2).

### Polymorphisms in the LRR domain determine L5 and L6 recognition specificity

**Figure 3:**
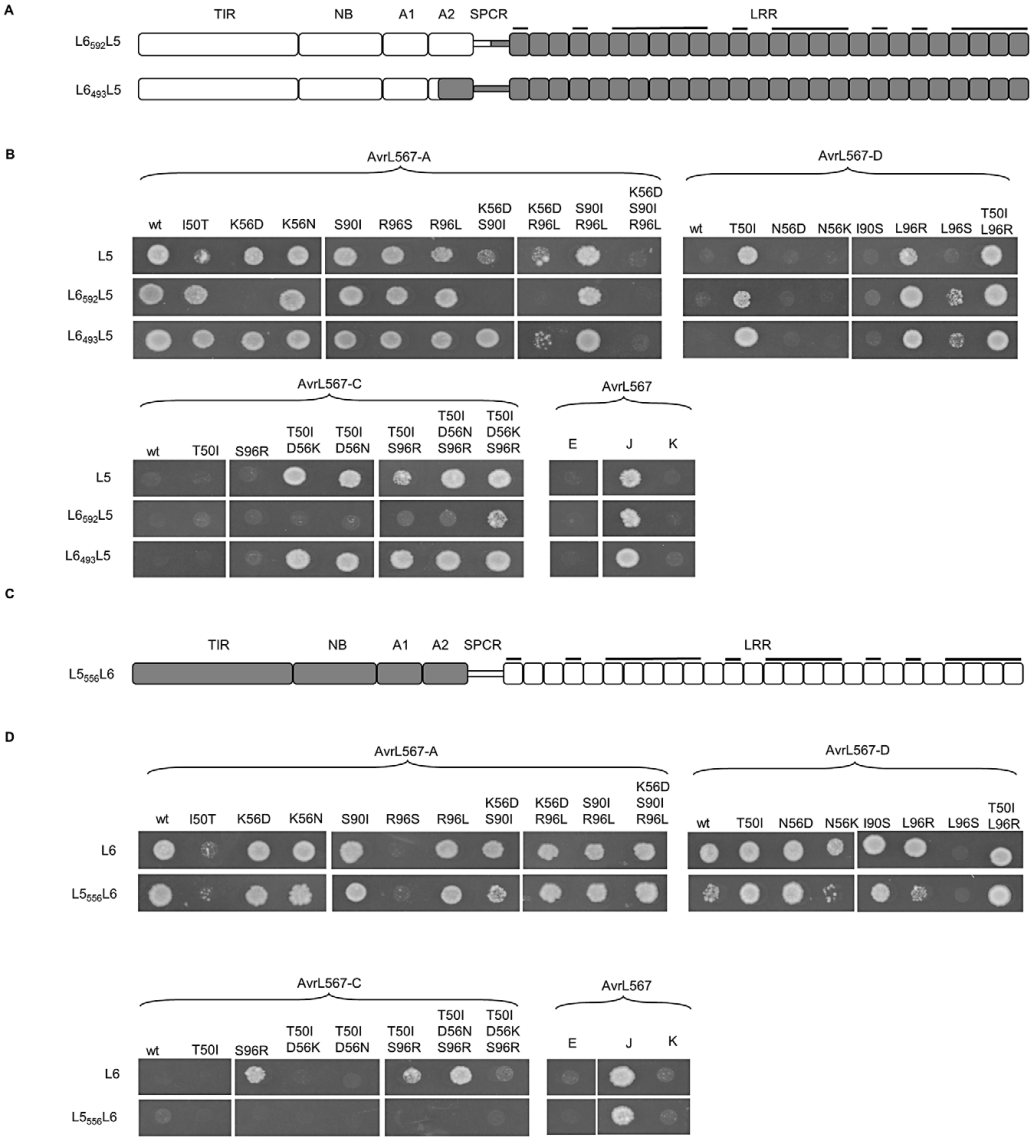
Polymorphisms found in the LRR domain determine L5 and L6 recognition specificity. **A and C.** Schematic diagrams of L6_592_L5, L6_493_L5 and L5_556_L6 chimeric proteins. Domains from L6 are shaded white and domains from L5 are shaded grey. LRR units with polymorphic residues in the β-strand/β-turn structure (xxLxLxx motif) are marked with black bars. **B and D.** Growth of yeast strain HF7c expressing L6_592_L5, L6_493_L5 or L5_556_L6 proteins fused to the GAL4 activation domain and wild-type (wt) and mutant AvrL567-A and -D proteins fused to the GAL4 DNA-binding domain. Cultures were grown on selective media lacking tryptophan, leucine and histidine and scored after 4 days.

The concentration of positively selected sites in the LRR domain of L proteins is consistent with the proposed role of this domain in recognition specificity. To test whether polymorphisms in the LRR domains of L5 and L6 are responsible for their different recognition specificity, we firstly generated chimeric proteins L6_592_L5 and L5_592_L6, in which the complete LRR domains of L5 and L6 are exchanged at an engineered *Avr*II restriction site in codons 592–593 (Figure S4). The introduction of this site causes a W to R amino acid change at position 592, but this substitution did not effect AvrL567-A or -D recognition by the modified L5 or L6 alleles (Figure S5) and is also found in the functional L9 protein. The chimeric proteins were tested for recognition of AvrL567 variants and mutants by the Y2H assay (Figure 3). Both recombinant proteins were well-expressed in yeast (Figure S2), but L5_592_L6 was non-functional in that it did not interact with either AvrL567-A or -D (Figure 5a). This may be related to the position of the exchange site within a seven-amino acid indel polymorphism (Figure S3). On the other hand, L6_592_L5 exhibited L5-like specificity, giving recognition of AvrL567-A but not -D, which indicated that this recognition pattern was determined by the LRR domain of L5 (Figure 3b). However, when tested against the extended set of AvrL567 mutants, L6_592_L5 recognized only a subset of the wild-type L5 repertoire, and failed to interact with AvrL567-A K56D, K56D/S90I and K56D/R96L mutants, and with most AvrL567-C gain-of function mutants (Figure 3b).

We therefore extended the region swapped between the alleles further towards the N-terminus. The chimera L6_493_L5, which contained the L5 LRR domain plus an additional three amino acid polymorphisms from the ARC2 domain and the entire wild-type spacer region (Figure 3a, S3), retained the full L5 recognition specificity across the pool of AvrL567 variants (Figure 3b). The only exceptions were a slightly enhanced interaction with the AvrL567-A I50T and K56D/S90I mutants, which only weakly interacted with L5, and a weak interaction with AvrL567-D L96S, which did not interact with L5. Similarly, the chimera L5_556_L6 that included the L6 LRR domain and the spacer region had L6-like recognition specificity when tested against wild-type AvrL567 variants and mutants (Figure 3c,d), although in some cases interactions were weaker than for L6 (AvrL567-D, AvrL567-D N56K and L96R and the AvrL567-C mutants). In conclusion, the data are consistent with the L5–L6 specificity differences being contributed by polymorphisms in the LRR domain and spacer region, but with the strength of the R:Avr protein interactions modulated somewhat by interactions between these regions and the N-terminal TIR-NB-ARC region.

### Polymorphisms found in the N- and C-terminal LRRs distinguish L5 and L6 specificity

**Figure 4:**
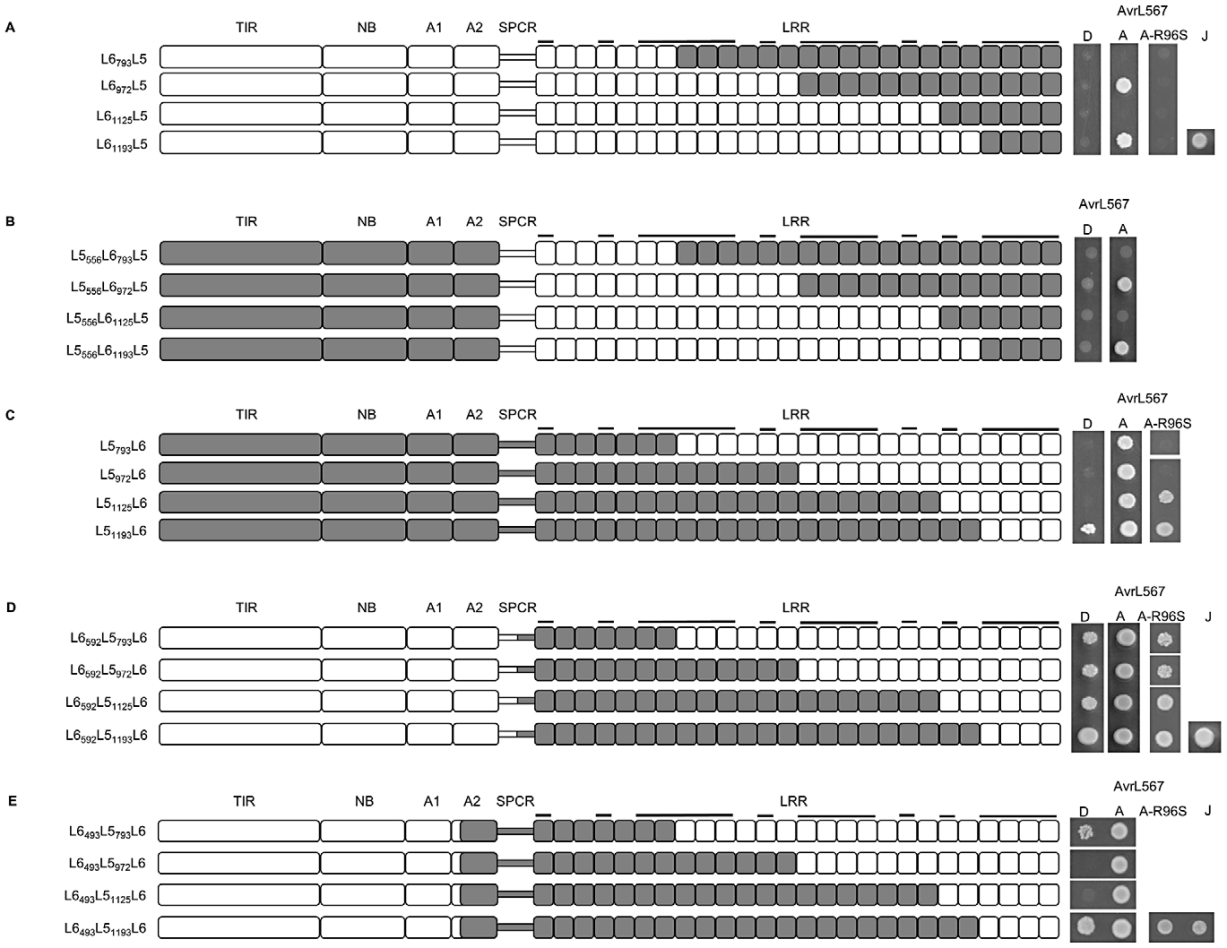
Polymorphisms found in the N- and C-terminal LRR units are critical for AvrL567 specificity. **A–E** left panel: Schematic diagram of chimeric L5–L6 proteins. Domains from L6 are shaded white and domains from L5 are shaded grey. LRR units with polymorphic residues in the β-strand/β-turn structure (xxLxLxx motif) are marked with black bars. **A–E** right panel: Growth of yeast strain HF7c expressing chimeric L5–L6 proteins fused to the GAL4 activation domain and AvrL567-A, -D, and -J proteins fused to the GAL4 DNA-binding domain. Cultures were grown on selective media lacking tryptophan, leucine and histidine and scored after 4 days.

The recognition repertoires conferred by the LRR domains of L5 and L6 can be qualitatively distinguished by their interactions with AvrL567-D (interacts with L6 but not L5) and the AvrL567-A mutant R96S (interacts with L5 but not L6) (Figure 1 and 3). To further understand the role of LRR domain polymorphisms in these differences, a series of L5–L6 chimeras with swaps within the LRR domain was generated. We designed swaps that would exchange groups of positively selected amino acids, as well as residues implicated in interaction in the docking-derived models of AvrL567 binding to a modelled L5 LRR structure presented by Wang *et al.* [45] (Figure S3). All the chimeric proteins were stably expressed in yeast (Figure S2) and were evaluated for interactions with AvrL567-A, -D and the AvrL567-A-R96S mutant (Figure 4).

These experiments allowed us to draw several inferences about residues controlling recognition specificity. Firstly, interaction with AvrL567-D, which discriminates the L6 specificity, requires L6 polymorphisms in the last four LRR units. Substitution of these four LRR units of L6 with the corresponding region of L5 (L6_1193_L5), abolished the interaction with AvrL567-D, but not -A (Figure 4a). This was also true for the same LRR exchange made in the context of the L5 TIR-NB-ARC (L5_556_L6_1193_L5; Figure 4b). Conversely, the reciprocal exchange in L5 (L5_1193_L6) allowed weak interaction with AvrL567-D (Figure 4c), suggesting that the L6 polymorphisms in the last four LRR units are both necessary and sufficient to confer AvrL567-D interaction in these proteins. Interestingly, this interaction was stronger when the L6 TIR-NB-ARC region was also present (L6_592_L5_1193_L6; Figure 4d). Indeed, the L6 TIR-NB-ARC region enhanced the recognition of AvrL567-D for all the chimeras containing the L6 C-terminal LRRs (compare Figures 4c and 4d).

Secondly, the ability to interact with AvrL567-A-R96S, which discriminates the L5 specificity, requires L5-specific polymorphisms in the first seven LRR units. L6_592_L5, containing the full-length L5 LRR domain, interacted with AvrL567-A-R96S (Figure 3), while proteins with chimeric L6-L5 LRR domains containing L6 N-terminal LRR polymorphisms (L6_793_L5, L6_972_L5, L6_1125_L5 and L6_1193_L5) did not (Figure 4a). Conversely, chimeras containing the reciprocal region from L5 swapped into the remainder of L6 (L6_592_L5_793_L6, L6_592_L5_972_L6, L6_592_L5_1125_L6, and L6_592_L5_1193_L6) acquired the capacity to interact with AvrL567-A-R96S (Figure 4d). Thus, the L5 polymorphisms found between residues 594 and 793 in L5 are necessary and sufficient to determine the AvrL567-A-R96S interaction in these proteins.

Intriguingly, two chimeric proteins (L5_793_L6 and L5_972_L6), which contain the critical 594-to-793 L5 residues along with the L5 TIR-NB-ARC domains did not interact with AvrL567-A-R96S (Figure 4c). As above, this suggests that the presence of L6 TIR-NB-ARC region is required for strong Avr protein interactions in proteins containing the L6 C-terminal LRRs. Neither L6_493_L5_972_L6 nor L6_493_L5_1125_L6 interacted with AvrL567-D (Figure 4e), suggesting an additional positive contribution to the strength of the interaction between these LRR chimeras and AvrL567-D may be attributed to the presence of one or more of the three L6-specific ARC2 polymorphisms (Figure S3).

We also observed that certain L6-L5 LRR chimeras lacked recognition function. For instance, swaps containing the N-terminal LRRs of L6 and the C-terminal LRRs of L5 gave rise to non-functional proteins when the junctions were made at positions 793 or 1125, but not at 972 or 1193 (Figure 4a and 4b). Similarly, proteins containing the N-terminal LRRs of L5 and the C-terminal LRRs of L6 gave rise to proteins with reduced functionality when the junctions were made at positions 793, 972 or 1125 (Figure 4c), although this could be overcome by the presence of polymorphisms found in the L6 TIR-NB-ARC region (compare swaps in Figure 4c, d and e). These observations suggest a requirement for specific, co-operative combinations of polymorphisms within the LRR domain to allow interaction with the corresponding ligand, consistent with the interaction occurring across a large surface area.

### Co-operative polymorphisms in TIR, ARC and LRR domains occur in L5 and L6, and are required for recognition function

**Figure 5:**
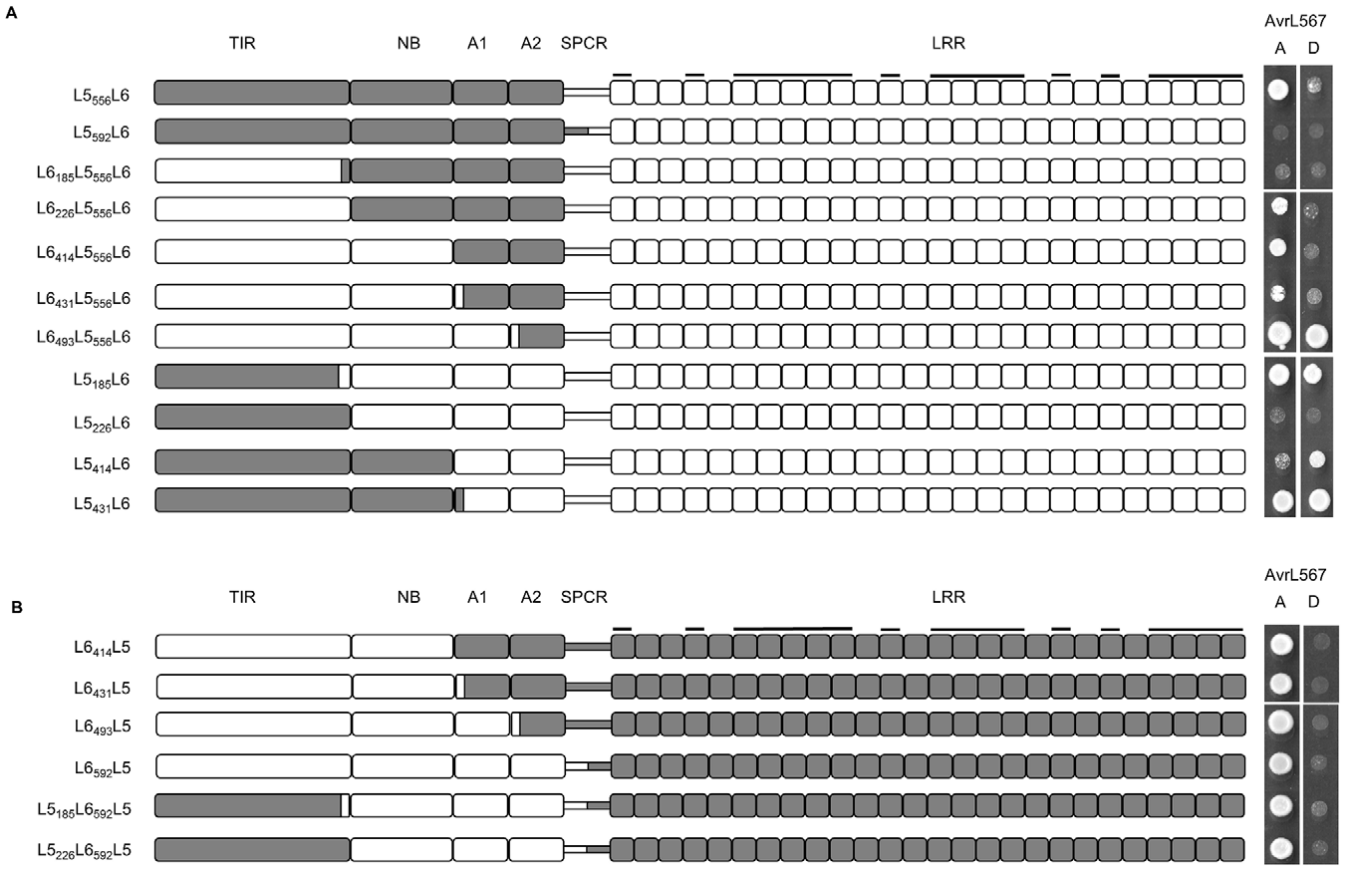
Polymorphisms found in the ARC domains contribute to L6 recognition strength. **A and B** left panel: Schematic diagram of chimeric L5–L6 proteins. Domains from L6 are shaded white and domains from L5 are shaded grey. LRR units with polymorphic residues in the β-strand/β-turn structure (xxLxLxx motif) are marked with black bars. **A and B** right panel: Growth of yeast strain HF7c expressing chimeric L5–L6 proteins fused to the GAL4 activation domain and AvrL567-A and -D proteins fused to the GAL4 DNA-binding domain. Cultures were grown on selective media lacking tryptophan, leucine and histidine and scored after 4 days.

Because the strength of AvrL567 interaction of several chimeric proteins appeared to be influenced by whether the TIR-NB-ARC region is derived from L5 or L6, we decided to examine the influence of polymorphisms in the N-terminal region on Avr protein interaction. A series of chimeras were generated in which various regions of the L6 TIR, NB, ARC1 and ARC2 domains were re-introduced into the L5_556_L6 protein, which exhibited L6-like specificity, but weaker AvrL567-D interaction, and tested for interaction with AvrL567-A and -D (Figure 5a). All chimeric proteins were stably expressed in yeast (Figure S2).

Introduction of increasing lengths of L6 sequence from the N-terminus (L6_185_L5_556_L6 to L6_431_L5_556_L6), including the TIR and NB regions, did not increase the interaction with AvrL567-D, but a further swap including the L6 ARC1 (L6_493_L5_556_L6) restored strong interaction with AvrL567-D. This suggested that one or more of the six amino acid polymorphisms between 447 and 484 (five in ARC1 and one in ARC2) were responsible for the reduced interaction. Consistent with this, inclusion of the ARC1 and ARC2 regions of L6 along with the L5 TIR-NB (L5_431_L6) also restored recognition of AvrL567-D, again implicating this region in the reduced interaction. Chimeric proteins representing the inverse swaps and including the L5 LRR domain (L6_414_L5, L6_431_L5, L6_493_L5, L6_592_L5, L5_185_L6_592_L5 and L5_226_L6_592_L5) did not interact with AvrL567-D but retained interaction with AvrL567-A (Figure 5b), similar to L5. This suggests that polymorphisms in the LRR domain of L6 are required to provide the specific recognition capacity to bind to AvrL567-D, while those in the ARC1/2 region may contribute to the strength of the interaction through intramolecular interactions.

**Figure 6:**
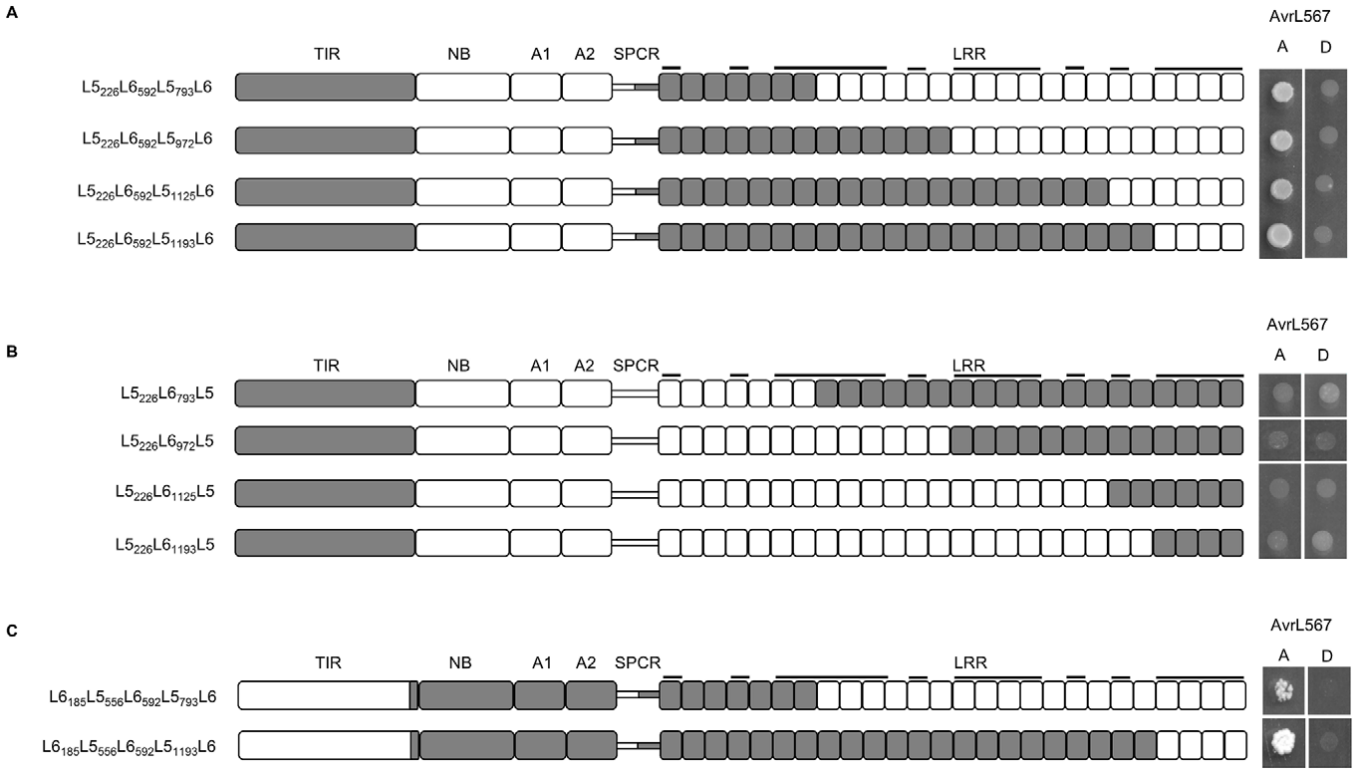
Polymorphisms in L5 co-vary between the TIR domain and the N-terminal LRRs. **A–C left panel:** Schematic diagram of chimeric L5–L6 proteins. Domains from L6 are shaded white and domains from L5 are shaded grey. LRR units with polymorphic residues in the β-strand/β-turn structure (xxLxLxx motif) are marked with black bars. **A–C right panel:** Growth of yeast strain HF7c expressing chimeric L5–L6 proteins fused to the GAL4 activation domain and AvrL567-A and -D proteins fused to the GAL4 DNA-binding domain. Cultures were grown on selective media lacking tryptophan, leucine and histidine and scored after 4 days.

Interestingly, some other swaps in the TIR-NB region also led to reduced Avr protein interaction. Notably, while the L5_185_L6 chimera was functional, L5_226_L6 did not interact with either AvrL567-A or -D. Similarly, L6_185_L5_556_L6 failed to interact with the Avr proteins, while L6_226_L5_556_L6 did interact with AvrL567-A. However, both the reciprocal swaps (L5_185_L6_592_L5 and L5_226_L6_592_L5, Figure 5b) interacted with AvrL567-A. This suggests that the two L5-derived amino acid polymorphisms in this region (E216 and L218) interfere with recognition in the context of the L6 LRR domain. We therefore tested a series of constructs containing chimeric L5–L6 LRR domains in the context of the L5_226_L6 protein, to determine which part of the L6 LRR domain mediates this incompatibility (Figure 6). Introduction of the seven N-terminal LRR units from L5 was sufficient to restore AvrL567 interaction in this protein (Figure 6a), while all chimeras containing this region from L6 failed to interact (Figure 6b). A similar pattern was observed for chimeras containing a hybrid L6_185_L5 TIR-NB junction (L6_185_L5_556_L6, L6_185_L5_556_L6_592_L5_793_L6 and L6_185_L5_556_L6_592_L5_1193_L6; Figure 5a and Figure 6c). This suggests that a negative interaction occurs between these TIR domain polymorphisms of L5 and polymorphic residues in the N-terminal region of the L6 LRR.

Even though several of these constructs contained all of the polymorphisms from L6 that are required for strong recognition of AvrL567-D, the presence of the hybrid L5–L6 TIR-NB junction appears to have prevented this interaction, for example in L6_185_L5_556_L6_592_L5_793_L6 and L6_185_L5_556_L6_592_L5_1193_L6 (Figure 6c).

### L6_493_L5_1193_L6 has a novel recognition specificity

As described above, chimeras L6_493_L5_1193_L6 and L6_592_L5_1193_L6, which contain the N-terminal L5 specificity region and the C-terminal L6-specificity region, represent a novel and expanded recognition specificity, in that they recognise both AvrL567-A-R96S and AvrL567-D, which distinguish L5 and L6 (Figure 4). We therefore tested these further against the larger set of AvrL567 variants and mutants and compared this to the recognition repertoires of L5, L6, L6_493_L5 and L6_592_L5 (Table 2 and Figure S6). The L6_592_L5_1193_L6 and L6_493_L5_1193_L6 recognition specificities were largely L6_592_L5-like and L6_493_L5-like, respectively, however both were L6-like in regards to interactions with AvrL567-D and its derived mutants. Overall, L6_493_L5_1193_L6 recognized more AvrL567 variants and mutants (23) than L6 (21), L6_493_L5 (20), L5 (19), L6_592_L5_1193_L6 (14), or L6_592_L5 (13) (Table 2).

**Table 2:** AvrL567 recognition repertoires of L5, L6, L6_592_L5, L6_592_L5_1193_L6, L6_493_L5, and L6_493_L5_1193_L6 as determined in the yeast-two-hybrid assay.

	AvrL567-A											AvrL567-D								AvrL567-C								AvrL567-		
											K56D															T50I	T50I			
									K56D	S90I	S90I								T50I				T50I	T50I	T50I	D56N	D56K			
	wt	I50T	K56D	K56N	S90I	R96S	R96L	S90I	R96L	R96L	R96L	wt	T50I	N56D	N56K	I90S	L96R	L96S	L96R	wt	T50I	S96R	D56K	D56N	S96R	S96R	S96R	E	J	K
**L5**	+	+/−	+	+	+	+	+	+/−	+/−	+	−	−	+	−	−	−	+	−	+	−	−	−	+	+	+/−	+	+	−	+	−
**L6**	+	+/−	+	+	+	−	+	+	+	+	+	+	+	+	+	+	+	−	+	−	−	+	−	−	+	+	−	−	+	−
**L6_592_L5**	+	+	−	+	+	+	+	−	−	+	−	−	+	−	−	−	+	+/−	+	−	−	−	−	−	−	−	+/−	−	+	−
**L6_592_L5_1193_L6**	+	+/−	−	++	+	+	+	−	−	+	−	+	+	−	+	+	+	−	+	−	−	−	−	−	−	−	−	−	+	−
**L6_493_L5**	+	+	+	+	+	+	+	+	+/−	+	−	−	+	−	−	−	+	+/−	+	−	−	−	+	+	+	+	+	−	+	−
**L6_493_L5_1193_L6**	+	+/−	+	+	+	+	+	+	+	+	+	+	+	−	+	+	+	−	+	−	−	−	+	+	+/−	+	+	−	+	−

−indicates no interaction, +indicates an interaction, +/−indicates a weak interaction.

**Figure 7:**
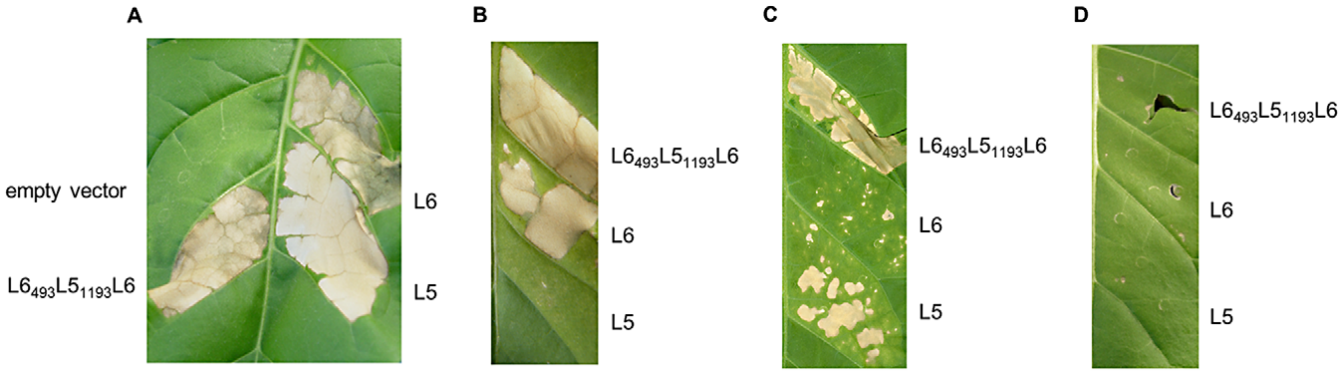
L6_493_L5_1193_L6 recognizes AvrL567-A and -D *in planta*. **A.** Transgenic tobacco W38 expressing AvrL567-A, 4 days after infiltration with *A. tumefaciens* strains carrying L5, L6 or L6_493_L5_1193_L6. **B.** W38 tobacco 4 days after co-infiltration with *A. tumefaciens* strains carrying L5, L6 or L6_493_L5_1193_L6 and AvrL567-D. **C.** W38 tobacco 4 days after co-infiltration with *A. tumefaciens* strains carrying L5, L6 or L6_493_L5_1193_L6 and AvrL567-A-R96S. **D.** W38 tobacco 4 days after co-infiltration with *A. tumefaciens* strains carrying L5, L6 or L6_493_L5_1193_L6 and an empty vector.

We further tested the L6_493_L5_1193_L6 chimera for its ability to trigger an AvrL567-dependent cell death *in planta* (Figure 7). *Agrobacterium*-mediated transient expression of the L5, L6 and L6_493_L5_1193_L6 cDNAs in transgenic tobacco also expressing AvrL567-A induced a strong HR-like cell death response (Figure 7a), confirming that the L6_493_L5_1193_L6 construct expresses a functional resistance protein. Co-expression of L6_493_L5_1193_L6 with AvrL567-D or AvrL567-A-R96S also induced an HR, while L5 induced cell death only with AvrL567-A-R96, and L6 only with AvrL567-D (Figure 7b,c), thus recapitulating the recognition specificity observed in yeast. No HR was observed when L6_493_L5_1193_L6 was expressed alone (Figure 7d), indicating that this chimera is not autoactive.

## Discussion

The L5/L6:AvrL567 system represents one of the most suitable models to characterize the specificity determinants of the R:Avr protein interaction, because there is a direct physical contact between the R and Avr proteins and the crystal structures of the Avr protein and part of the R protein have been determined [4], [45]. We had previously shown that certain surface-exposed residues of AvrL567 were important for their recognition by L5 and L6 [45]. Here we have further confirmed the roles of these positions in recognition in different AvrL567 protein contexts and have shown by analysis of single, double and triple mutations that they make additive and cumulative contributions to recognition specificity. We also demonstrated the role of the LRR domain in determining the specificity of L5 and L6 towards AvrL567 variants, and found that polymorphisms in the C- and N- terminal LRRs are required for recognition specificity. The concentration of polymorphic sites at the two ends of the LRR domain suggests that the AvrL567 ligand may bind between the two ends of the LRR horseshoe structure, consistent with an *in silico* docking model [45]. This further confirms that multiple, additive contact points occur between AvrL567 variants and either L5 or L6. However, we also observed that recognition of AvrL567 is affected by co-operative polymorphisms within the LRR domain as well as the in TIR and ARC domains; this suggests that these residues are also involved in intramolecular interactions that influence ligand accessibility. We propose that ligand binding occurs in competition with intramolecular interactions that serve to maintain the protein in an inactive signalling state.

### Additive effects of polymorphic AvrL567 residues in interactions with L resistance proteins

Mutational analysis shows that multiple sites are involved in the interaction between AvrL567 variants and L5 and L6, and that L5 and L6 have different specificity requirements at these positions (Figure 1). The additive nature of the interactions is shown by the effects of double and triple mutations in the AvrL567 proteins on recognition. For instance, while single mutations at positions 56, 90 and 96 do not disrupt recognition by L5, triple substitution abolishes L5 recognition. However, these changes do not disrupt recognition by L6, highlighting the different sequence requirements of the two resistance proteins. Conversely, with the exception AvrL567-C-S96R (which is weakly recognised by L6), the virulence allele AvrL567-C required at least two to three mutations in combination to allow full recognition by L5 or L6. Again, the requirements for the two resistance proteins were different. A double substitution at positions 50 and 56 was sufficient for L5 recognition, while full L6 recognition required a further substitution at position 96, and showed a requirement for an asparagines residue at position 56 rather than lysine.

Previously, Wang *et al.* [45] demonstrated that T50 in AvrL567 destabilizes interactions with L5 and L6 and our data further confirm that this residue is particularly important for recognition. For instance, the T50I substitution has a strong positive effect in AvrL567-E on stabilizing interactions with L5 and L6 (Figure 1). AvrL567-E and -J differ by only two polymorphisms (H26D and T50I), but we previously found that the N-terminal region consisting of amino acids 26–37 of AvrL567-A could be deleted without affecting recognition [43]. Therefore, the T50I polymorphism is the critical residue that differentiates AvrL567-E and -J recognition specificities. The importance of position 50 can also be observed by the contribution a T50I substitution makes to allow L5 recognition of AvrL567-C when paired with D56N, D56K, or S96R substitutions, and to allow L6 to recognize AvrL567-C when associated with D56N and S96R in a triple substitution (Figure 1).

The presence of a D residue at position 56 had a small negative effect on interactions with L5 but not L6 [45], and we observed that this effect is much stronger when combined with either, or both, S90I and R96L in AvrL567-A. Similarly, neither S90I or R96L substitutions (Table 1), nor the double S90I/R96L substitution in AvrL567-A compromise recognition by L5; however, I90 and L96 both have stronger negative effects on interactions with L5 when combined with D56, and completely disrupt L5 recognition when all three substitutions are present in AvrL567-A (Figure 1). Interestingly, in the context of AvrL567-C, K56 has a negative effect on recognition by L6 but not L5, as the triple T50I/D56N/S96R substitution in AvrL567-C is recognized by both L5 and L6 in yeast, whereas the triple T50I/D56K/S96R substitution in AvrL567-C is only recognized by L5.

Position 96 in AvrL567 is important for interactions with both L5 and L6, with R at this position favouring interactions with L5, and an S disrupting interactions with L6 (Figure 1 and [45]). For instance, the R96S substitution in AvrL567-A destabilizes interactions with L6, whereas the reciprocal S96R substitution improves interaction of AvrL567-C with L6 both individually, and in combination with T50I or T50I/D56N substitutions, and with L5 when combined with the T50I, T50I/D56N or T50I/D56K substitutions. However, as with other polymorphisms in AvrL567, the disruptive effect of S96 on L6 recognition is context-dependent, as both AvrL567-E and –J contain this polymorphism while maintaining interactions with L6.

Collectively, these data indicate that L5 and L6 interact with AvrL567 through multiple amino acid contact points, and support the hypothesis that recognition is mediated in an additive manner by the cumulative composition and context of their amino acid sequences.

### N and C-terminal LRR polymorphisms contribute to AvrL567 recognition

Chimeric L5–L6 proteins containing reciprocally swapped LRR domains showed AvrL567 interaction specificities consistent with the origin of the LRR domain (Figure 3), although some interactions were weaker than in the wild-type proteins. Positively selected amino acid sites in L alleles cluster at the N and C-terminal regions of the LRR (Figure 2), and docking analysis of AvrL567 to the modelled L5 LRR domain [45] suggested that the most likely binding site of AvrL567 is between the two ends of the LRR with most potential contact points in these N- and C-terminal regions. This hypothesis is supported by analysis of LRR chimeras (Figure 4), which showed that 13 polymorphisms in the last four LRRs of L6 are required for the recognition of AvrL567-D, while polymorphisms in the first seven LRRs of L5 are required for recognition of AvrL567-A-R96S. Some internal LRR fusions (eg. L6_793_L5 and L6_1125_L5; Figure 4a) fail to interact with AvrL567 variants, or show a reduced interaction repertoire (eg. L6_493_L5_972_L6, L6_493_L5_1125_L6; Figure 4e), suggesting that surfaces involved in specific interactions may have been disrupted by fusions at these junctions.

Previously, Ellis *et al.* [41] showed that polymorphisms between L6 and L11 in the last three LRRs (24, 25 and 26) are important for L6 recognition of AvrL567. The L6L11RV chimera differs from L6 by 11 polymorphisms in these three LRR units, and recognizes only AvrL567-J, whereas L6L11B2, with two additional polymorphisms in LRR 23, does not interact with any AvrL567 variant (Figure S7). L5 is quite similar in sequence to L11 in this region, but a similar L6-L5 exchange in this region (L6_1193_L5) maintained interaction with many AvrL567 variants, including -A and -J (Figure 4). Comparison of the C-terminal sequences of these chimeras (Figure S7) narrows the L11 polymorphisms responsible for these difference down to R1220 and K1222 in LRR 24 – contributing to the loss of Avr recognition (other than -J) in L6L11RV, and V1196 leading to the loss of -J recognition in L6L11B2, because these are the only polymorphisms unique to L11. It is important to note that these domain-swap experiments only examine the roles of polymorphic residues in determining recognition specificity and do not address the role of shared residues in AvrL567 interaction. Indeed, docking analysis identified 31 residues common to L5 and L6 that may be involved in protein contacts.

Because chimeras with the N-terminal LRRs of L5 and C-terminal LRRs of L6 can interact with AvrL567-A, -A-R96S and -D, and reciprocal chimeras also retain interaction with at least AvrL567-A (Figure 4), we conclude that binding of AvrL567 to L5 and L6 occurs in the same basic orientation. The ability of the L5 N-terminal LRRs to allow binding to AvrL567-A-R96S may suggest that the AvrL567 surface region containing this residue makes contact with the N-terminal LRR region.

### Co-operative interactions between TIR, ARC and LRR domains influence AvrL567 binding

Although in general domain-swaps involving the full LRR domain showed the expected specificity for the source of the LRR, some chimeras had weak or no interactions with AvrL567 variants (Figure 3). Because all of the polymorphisms found in L5 and L6 are compatible with AvrL567 interaction in their native context, this suggests that certain polymorphisms in L5 and L6 N-terminal regions occur in specific, co-operative combinations that are required for recognition function. We observed such co-adaptation between the spacer region and the LRR domain, between the ARC domains and the C-terminal region of the LRR domain, and between the TIR and LRR domains. While both L6_493_L5 and L6_592_L5 exhibited L5-like specificity (Figure 3), when tested on the wider set of AvrL567 mutants, L6_592_L5 recognised only a subset of the wild-type L5 repertoire. This suggests that some or all of the nine L5-specific amino acids in the ARC2 and spacer region (Figure S3) are important for optimal recognition. Six of these residues represent a small insertional polymorphism in the spacer region, which may influence the relative positioning of the L5 LRR domain with respect to the rest of the protein. A wild-type spacer region is probably also required for L6 to adopt a functionally competent state, because an exchange within this indel in L5_592_L6 resulted in a non-functional protein, while L5_556_L6 (in which the exchange occurs before this indel) showed an L6-like recognition repertoire (Figure 3). Given its proximity to the LRR domain, it is possible that the spacer region also participates directly in AvrL567 binding.

On the other hand, the observed co-adaptation between polymorphisms at the C-terminus of the TIR domain and the N-terminus of the LRR domain, and between the ARC and LRR domains, likely reflects an indirect effect on ligand affinity as a result of intramolecular interactions that obscure the ligand-binding site, rather than a direct effect on binding specificity. Such general effects on ligand accessibility would be expected to manifest themselves particularly in the case of those interactions that are close to the threshold of detection in Y2H assays. Sequence exchanges involving regions of the TIR domain suggested that two L5-derived amino acid polymorphisms in this region (E216 and L218) interfere with recognition in the context of the L6 LRR. However, the TIR domain is not required for L6-AvrL567 interaction, and these two residues are exposed on the surface of the TIR domain structure in a region implicated in negative regulation of L6 through intramolecular interactions [26]. Indeed, TIR domain residues that are polymorphic between L6 and L7 also play a role in AvrL567 interaction and are responsible for the weak resistance phenotype of L7 [26], [30]. Likewise, polymorphisms in the ARC1 and ARC2 domains of L6 strengthen AvrL567-D recognition, conferred by the polymorphisms found in the last four LRRs of L6 (Figure 5). Again, this appears to be a general ligand affinity effect, because swapping the L6 ARC domains into L5 does not generate recognition of AvrL567-D (Figure 5b). Furthermore, the presence of the L6 TIR-NB-ARC region also strengthens interactions with AvrL567-A-R96S, mediated by polymorphisms found in the first seven LRRs of L5 (Figure 4c and d). We previously showed that a P-loop mutation (K271M) in L6, which would prevent nucleotide binding, also disrupted interaction with AvrL567 [16], consistent with the idea that interactions between the NB-ARC and LRR are required to support ligand binding.

Previous experiments in other systems have also demonstrated intramolecular interactions between R protein domains that are important for function. The CC, NB-ARC and LRR domains of potato Rx can interact and functionally complement each other when expressed as separate polypeptides [47]. Likewise, domain swaps have also implicated co-adaptation between domains of Rx, Mi-1.2 and I-2 in tomato, and Pm3 in wheat [34], [48], [49], [50]. It has been suggested that ARC1 functions as a molecular scaffold, forming intramolecular interactions with the LRR domain, and that signal perception disrupts these interactions [34]. Subsequently, ARC2 may transduce this effect into defence protein activation [50]. However, these experiments do not distinguish between effects on ligand recognition, protein activation or downstream signalling. Our data on AvrL567 binding by L5/L6 recombinants indicate that these intramolecular interactions can have direct effects on ligand binding. This suggests a model of R protein activation in which ligand binding occurs in direct competition with intramolecular interactions, which presumably maintain the resting protein in an inactive signalling state. Rather than Avr binding directly destabilising intramolecular interactions, it is possible that R proteins exist in an equilibrium between active and inactive states, with the Avr protein preferentially binding to and stabilising the active state to induce signalling. This competition provides a mechanism for signalling activation, as well as for fine-tuning the triggering of the response. Weak R:Avr interactions require a very delicate trigger if they are to induce effective resistance, but this would come at a cost of increased autoactivity of the R protein. Conversely, stronger Avr interactions could compete with more stable inhibitory intramolecular interactions.

**Figure 8:**
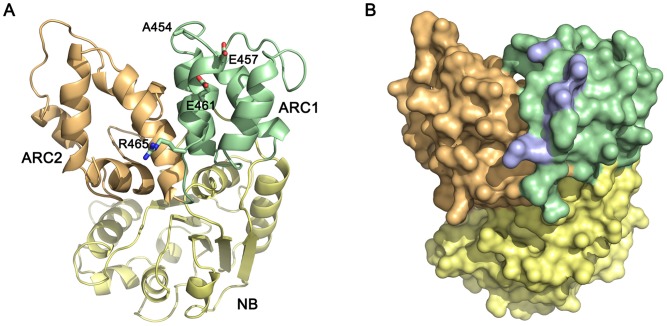
Homology model of the L6 NB-ARC domain. A homology model of the L6 NB-ARC domain was prepared using Modeller [51]. The NB sub-domain is coloured in yellow, the ARC1 sub-domain is coloured in green, and the ARC2 sub-domain is coloured in orange. **A.** Cartoon representation of the NB-ARC domain with the polymorphic residues A454, E457, E461 and R465 represented as sticks. **B.** Surface representation of the NB-ARC domain with the polymorphic residues in **A** highlighted in blue. The molecule is oriented as in **B.** The figure was prepared using PYMOL (http://www.pymol.org).

To visualize structurally the L protein regions involved in AvrL567 recognition, we prepared a homology model of the L6 NB-ARC domain with the program Modeller [51] using the multiple sequence alignment of NB-ARC domains from different R proteins published in van Ooijen *et al.* [11] and the crystal structure of APAF-1 as a template [52]. In the model, four of the L6 polymorphisms involved in strengthening interactions with AvrL567-D (A454, E457, E461, and R465) map to a solvent exposed region in the last α-helix of the ARC1 domain (Figure 8). This α-helix (H8 of HD1), is part of the ARC1-ARC2 linker region, found in APAF-1 and CED-4, that undergoes a drastic change during the switch from closed to open states [9]. Therefore in L6, these residues are positioned appropriately to be involved in either, or both, inter- and intramolecular interactions, and may provide a putative link between effector perception and hypothetical conformational changes that lead to the open state. Likewise, Brunner *et al.* [48] found that polymorphic residues in the ARC2 domain that disrupt Pm3 function appear to be concentrated on one side of the ARC2 domain, and are largely solvent exposed, suggesting that they may be involved in intra- or intermolecular interactions.

### Novel specificity generated by *in vitro* sequence exchange - a pathway for R gene engineering

In the course of this chimeric protein analysis we generated some recombinant proteins that exhibited novel and expanded recognition specificities, through bringing together unique combinations of polymorphic sites. One of these (L6_493_L5_1193_L6) was shown to function to induce an HR in tobacco, recapitulating the expanded recognition observed in Y2H assays. This correspondence between Avr:R interaction in yeast and HR induction *in planta*, now observed across a large number of L-AvrL567 pairwise combinations (Figure 1 and 7; [16], [45]) is consistent with the hypothesis of ligand interaction triggering signalling through competition with inhibitory intramolecular interactions. This suggests that recombination of existing polymorphisms through sequence exchanges is a powerful method for both generating changes in recognition specificity and fine-tuning the strength of defense response during evolution of resistance genes. This mechanism, along with induced mutation, may be adapted to engineering novel resistance genes that can be deployed in agriculture [53]. This process may require not only changes to LRR domain to generate new binding specificities, but also concomitant changes in N-terminal domains to optimise the defense signalling output.

## Materials and Methods

### Site-directed mutagenesis, construction of recombinant genes and yeast-two-hybrid analyses

Site-directed mutants of AvrL567 were constructed using the Gene-Tailor kit (Stratagene) according to the manufacturer's instructions. Chimeric L5–L6 proteins were constructed using native and introduced restriction sites, and/or by PCR-based fusion of overlapping sequences as described in Text S1 and Figure S5. Chimeric proteins were either constructed directly in pGADT7, or were sub-cloned in pBSK prior to construction in pGADT7. All constructs were checked by restriction enzyme digests and DNA sequencing. GAL4-binding domain (BD) fusions, and transcriptional activation domain (AD) fusions to L5, L6, L5–L6 chimeric proteins and to AvrL567 mutants and variants, were prepared in the pGBT9 and PGADT7 vectors (Clontech), as described [16], [45]. Yeast transformation, lacZ and His growth assays were performed as described in the Yeast Protocols Handbook (Clontech). Yeast proteins were extracted by the trichloro-acetic acid method, separated by SDS/PAGE, and transferred to nitrocellulose membranes (Pall) by electroblotting. Membranes were blocked with 5% skim milk and probed with anti-HA mouse monoclonal antibodies (Roche), followed by goat anti-mouse antibodies conjugated with horseradish peroxidase (Pierce). Labeling was detected with the SuperSignal West Pico chemiluminescence kit (Pierce).

### Transient expression assay

DNA constructs encoding AvrL567 proteins lacking the signal peptide, or full-length L5, L6 or L6_493_L5_1193_L6 cDNAs, were cloned into the binary vector pTNotTReg between the cauliflower mosaic virus 35S promoter and ocs terminator sequences. *Agrobacterium tumefaciens* (GV3101-pMP90) cells containing these constructs were grown for 36 h at 28°C in LB media supplemented with appropriate antibiotic selections. Cells were pelleted, resuspended in infiltration medium (10 mM MgCl_2_, 200 µM acetosyringone), adjusted to OD_600 nm_ = 1 and incubated for 2 h at room temperature. Resuspended cells were infiltrated with a 1-mL needleless syringe into the leaves of near isogenic lines of flax plants containing L5, L6 (cv. Bison) or L6L11RV (cv. Ward), or into leaves of 3-week-old tobacco plants (W38). Transgenic tobacco expressing AvrL567-A was described by Dodds *et al.* [42].

### Detection of positive selection

The non-synonymous/synonymous rate ratio parameter ω was estimated using the program CODEML [54] in phylogenetic analysis by ML v. 4.2. Tests for positive selection were performed using the site class models that estimate ω for amino acid sites. Neutral sites have ω = 1, those under purifying selection have ω<1, and those under positive selection have ω>1 [55]. Likelihood ratio tests were performed using lnL values from the models M7 and M8 by comparing the test statistic 2ΔlnL = 2(lnL_M7_−lnL_M8_), with the χ^2^ distribution (d.f. = 2). For model M8, the empirical Bayes [55] procedure estimated the mean ω-value for each codon site, and the posterior probability that the site is under positive selection.

## Supporting Information

Figure S1 **Mutational analysis of AvrL567-D interactions with L5 and L6.** Upper Panels: β-galactosidase activity of yeast strain SFY526 expressing the GAL4 DNA-binding domain fused to the L5 and L6 proteins along with the corresponding GAL4 activation domain fused to AvrL567-D variants. Bottom Panels: growth of yeast strain HF7c expressing the same protein fusion constructs on selective –W,L,H plates after 4 days.
(TIF)

Figure S2 **Immunoblot detection of fusion proteins expressed in yeast.** Protein extracts from yeast strains HF7c expressing **A.** AvrL567::GAL4-AD, and **B.** chimeric L5–L6::GAL4-AD fusion proteins were separated by SDS-PAGE, blotted onto nitrocellulose membranes and detected by anti-hemagglutinin mAbs. Positions and sizes of proteins molecular mass standards are indicated.
(TIF)

Figure S3 **Amino acid alignment of polymorphic residues of L5, L6 and all L5–L6 chimeras used in this study.** Residues from L5 are coloured blue, residues from L6 are coloured red. Amino acid position numbers are indicated above the alignment and within the LRR domain residues occurring in the β-turn and β-strand regions are numbered by the repeat that they occur in (1–26). Black plus sign indicates a residue under positive selection (*p*>0.95), red plus sign indicates a residue under positive selection (*p*>0.99). i indicates a residue involved in protein-protein interactions according to the *in silico* docking models of L5 and AvrL567-A [45].
(TIF)

Figure S4 **Selected primers (black) and restriction sites (blue) used in the construction of chimeric L5–L6 proteins.** See Text S1 for details.
(TIF)

Figure S5 **Growth of yeast strain HF7c expressing GAL4 DNA-binding domain fused to the L5_592_L5 and L6_592_L6 proteins along with the corresponding GAL4 activation domain fused to AvrL567-A or -D on selective –W,L,H plates after 4 days.** L5_592_L5 and L L6_592_L6 are identical to L5 and L6, respectively, except for a W593R substitution resulting from an engineered *Avr*II restriction site introduced to facilitate the generation of LRR domain swaps.
(TIF)

Figure S6 **Recognition repertoire of L6_592_L5_1193_L6 and L6_493_L5_1193_L6.** Growth of yeast strain HF7c expressing GAL4 DNA-binding domain fused to the L6_592_L5_1193_L6 and L6_493_L5_1193_L6 proteins along with the corresponding GAL4 activation domain fused to AvrL567 proteins on selective –W,L,H plates after 4 days.
(TIF)

Figure S7 **Polymorphic amino acid residues found in the LRR domain of chimeric L5–L6 and L6–L11** [**56**] **resistance proteins.** Residues from L5 are shaded blue, those from L6 are shaded red, and those from L11 are shaded green. LRR subunits with polymorphic residues in the β-strand/β-strand structure (xxLxLxx motif) are marked with black bars and are numbered below. All residues are listed below the corresponding L5 residues.
(TIF)

Table S1 **Log-likelihoods (lnL), Log-likelihoods ratio test (LRT), and estimates of the model parameters (κ: transition/transversion rate ratio; parameters of the beta distribution:** ***p*** **and** ***q*** **;** ***p1:*** **the fraction of sites estimated to fall within the class of sites ω>1, and the mean value (ω) for that class) for the models M7 and M8 in CODEML.**
(TIF)

Text S1 **Supplementary experimental procedures.**
(DOC)
