# Supplementary figures and images for: Intramolecular Interaction Influences Binding of the Flax L5 and L6 Resistance Proteins to their AvrL567 Ligands
Source: PLoS Pathog. 2012 Nov 29;8(11):e1003004. doi: 10.1371/journal.ppat.1003004 (PMC3510248; doi:10.1371/journal.ppat.1003004)

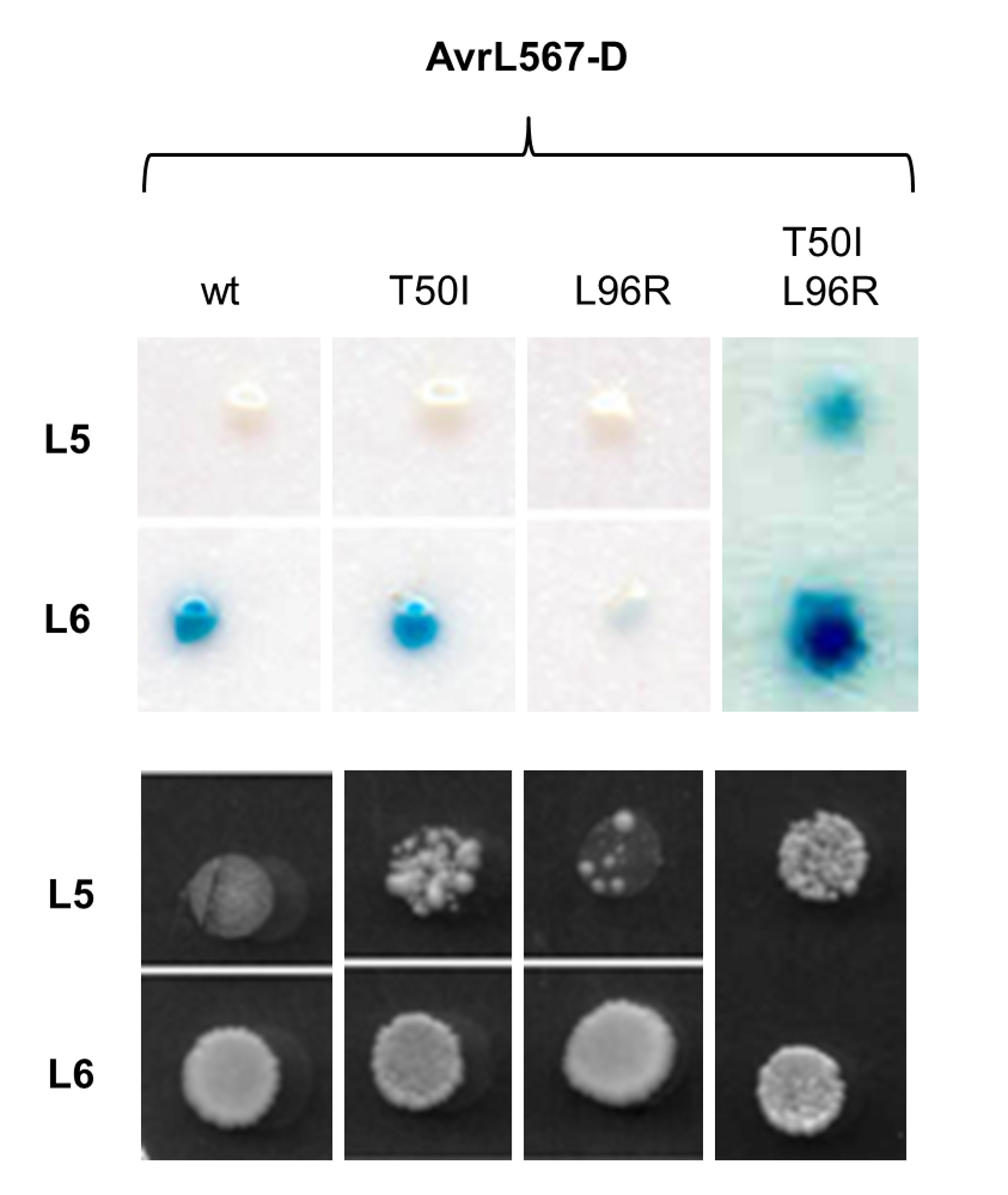

Supplement: Figure S1 — Mutational analysis of AvrL567-D interactions with L5 and L6. Upper Panels: β-galactosidase activity of yeast strain SFY526 expressing the GAL4 DNA-binding domain fused to the L5 and L6 proteins along with the corresponding GAL4 activation domain fused to AvrL567-D variants. Bottom Panels: growth of yeast strain HF7c expressing the same protein fusion constructs on selective –W,L,H plates after 4 days. (TIF) [file ppat.1003004.s001.tif]

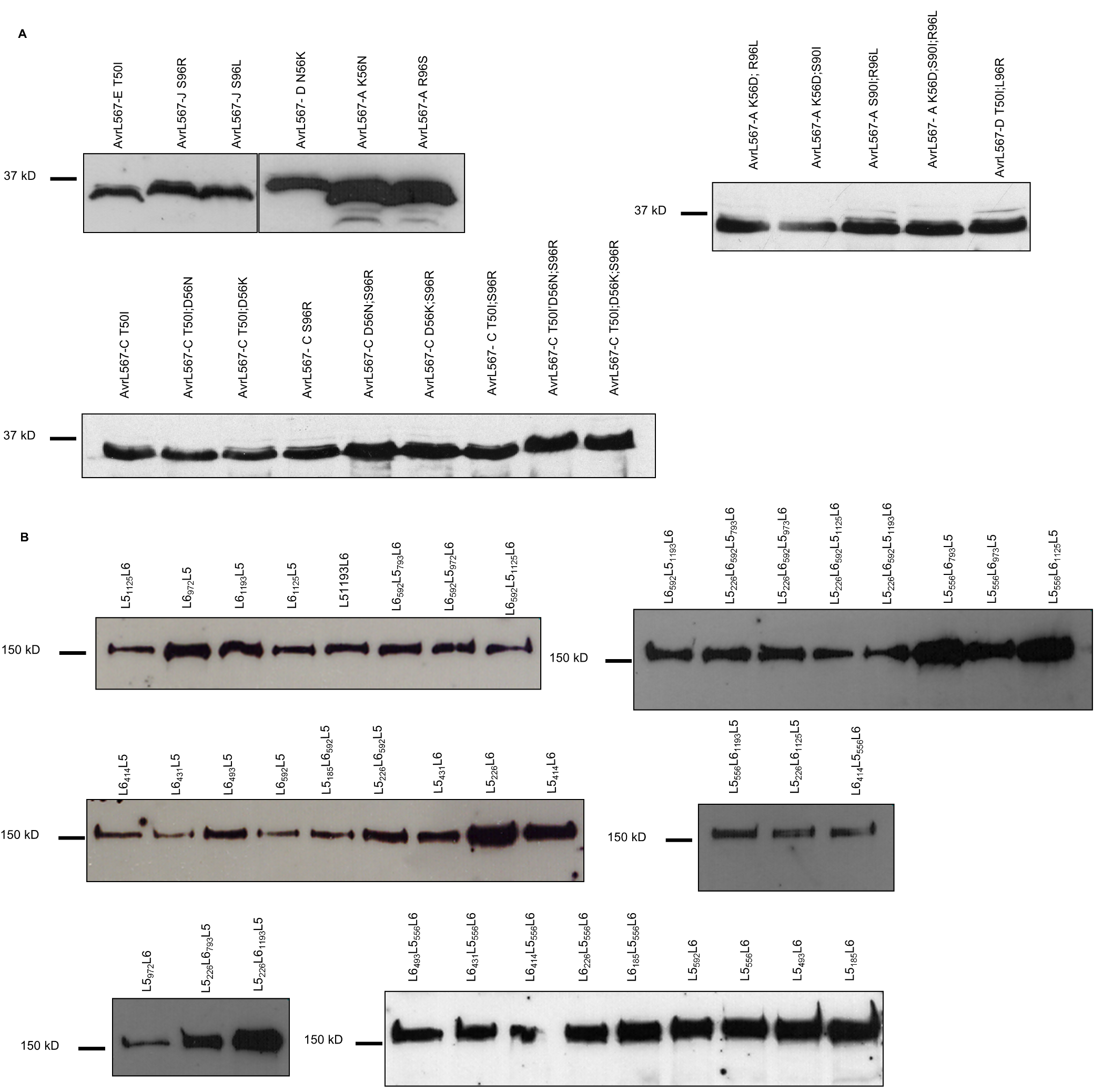

Supplement: Figure S2 — Immunoblot detection of fusion proteins expressed in yeast. Protein extracts from yeast strains HF7c expressing A. AvrL567::GAL4-AD, and B. chimeric L5–L6::GAL4-AD fusion proteins were separated by SDS-PAGE, blotted onto nitrocellulose membranes and detected by anti-hemagglutinin mAbs. Positions and sizes of proteins molecular mass standards are indicated. (TIF) [file ppat.1003004.s002.tif]

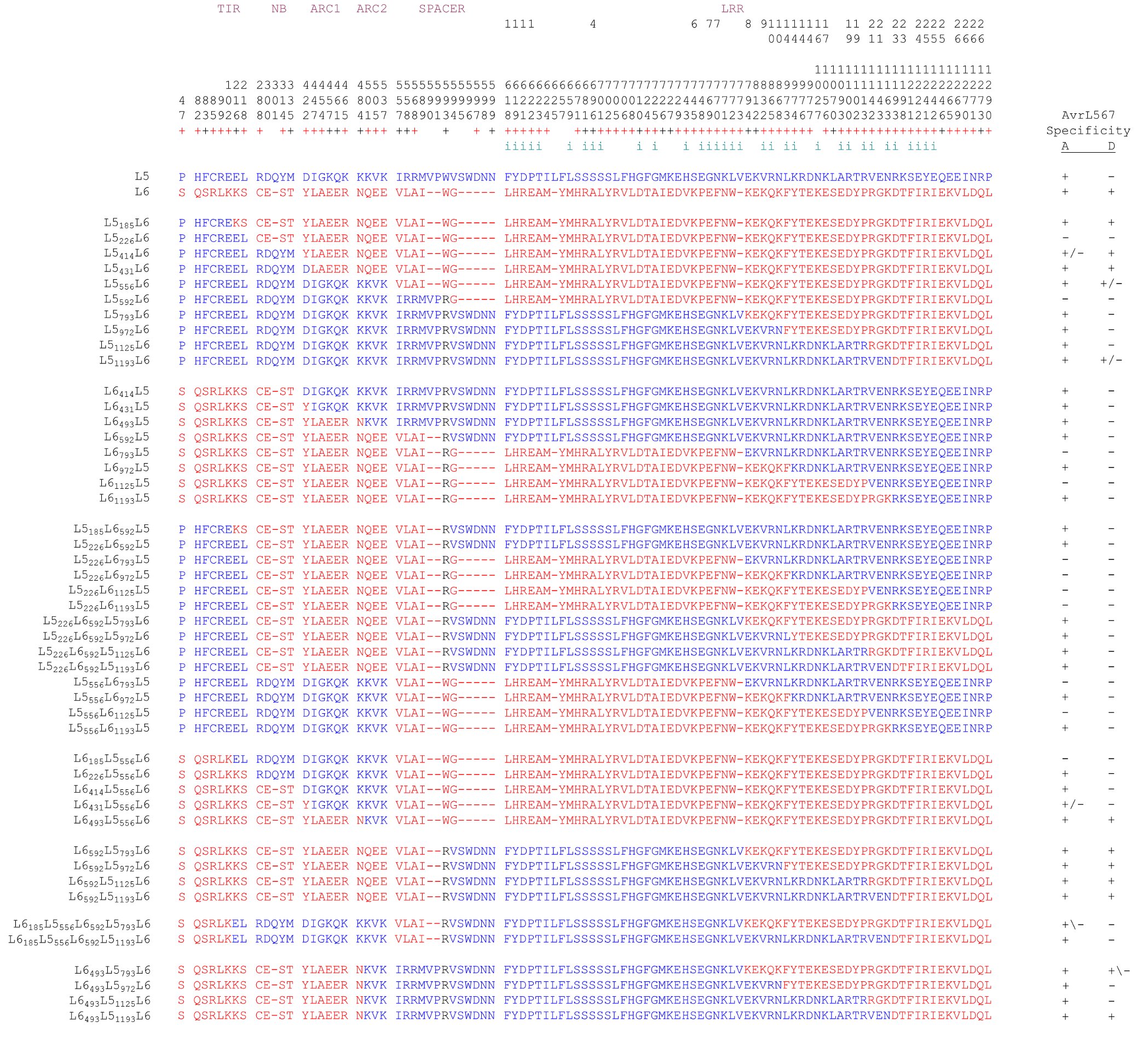

Supplement: Figure S3 — Amino acid alignment of polymorphic residues of L5, L6 and all L5–L6 chimeras used in this study. Residues from L5 are coloured blue, residues from L6 are coloured red. Amino acid position numbers are indicated above the alignment and within the LRR domain residues occurring in the β-turn and β-strand regions are numbered by the repeat that they occur in (1–26). Black plus sign indicates a residue under positive selection (p>0.95), red plus sign indicates a residue under positive selection (p>0.99). i indicates a residue involved in protein-protein interactions according to the in silico docking models of L5 and AvrL567-A [45]. (TIF) [file ppat.1003004.s003.tif]

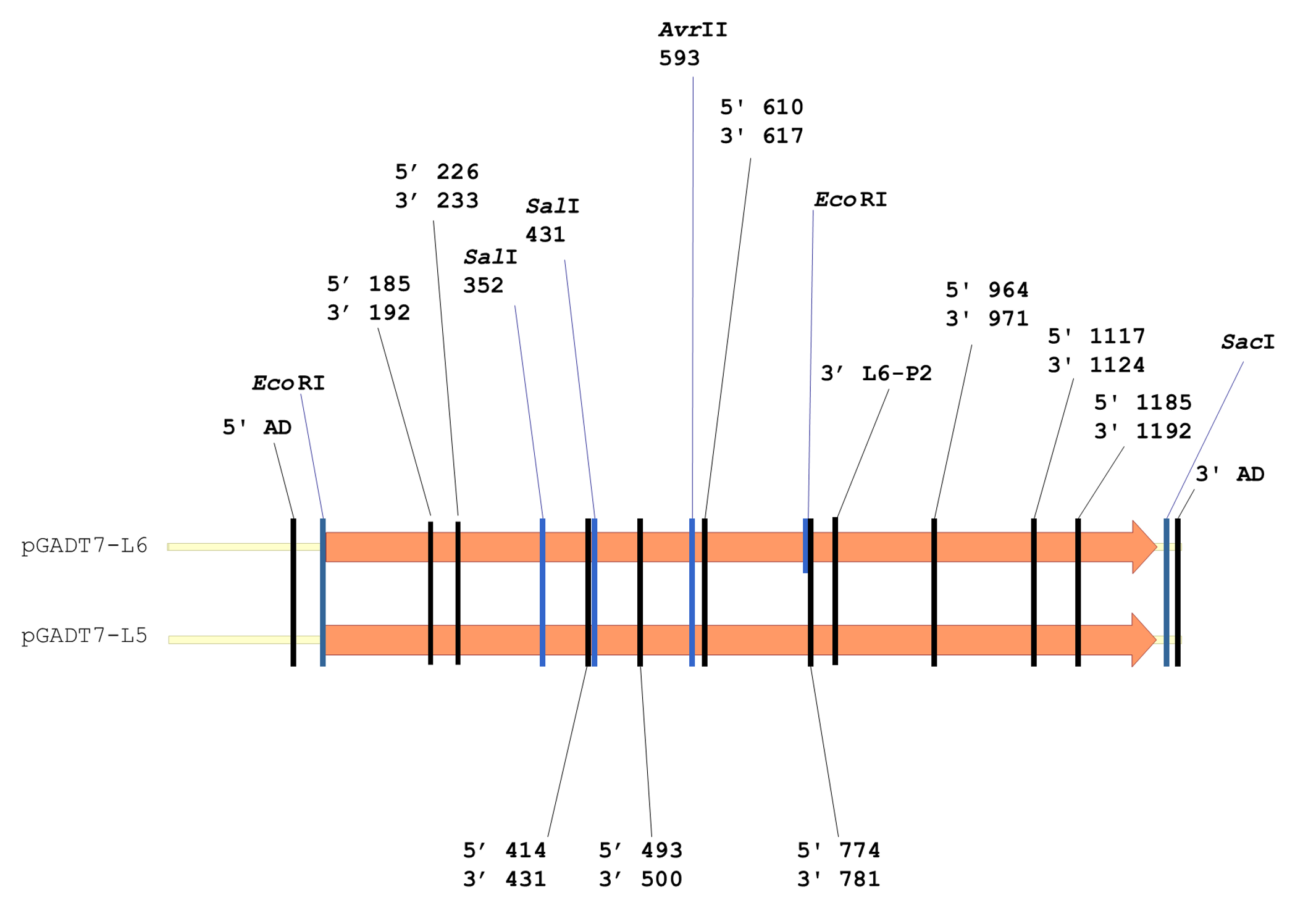

Supplement: Figure S4 — Selected primers (black) and restriction sites (blue) used in the construction of chimeric L5–L6 proteins. See Text S1 for details. (TIF) [file ppat.1003004.s004.tif]

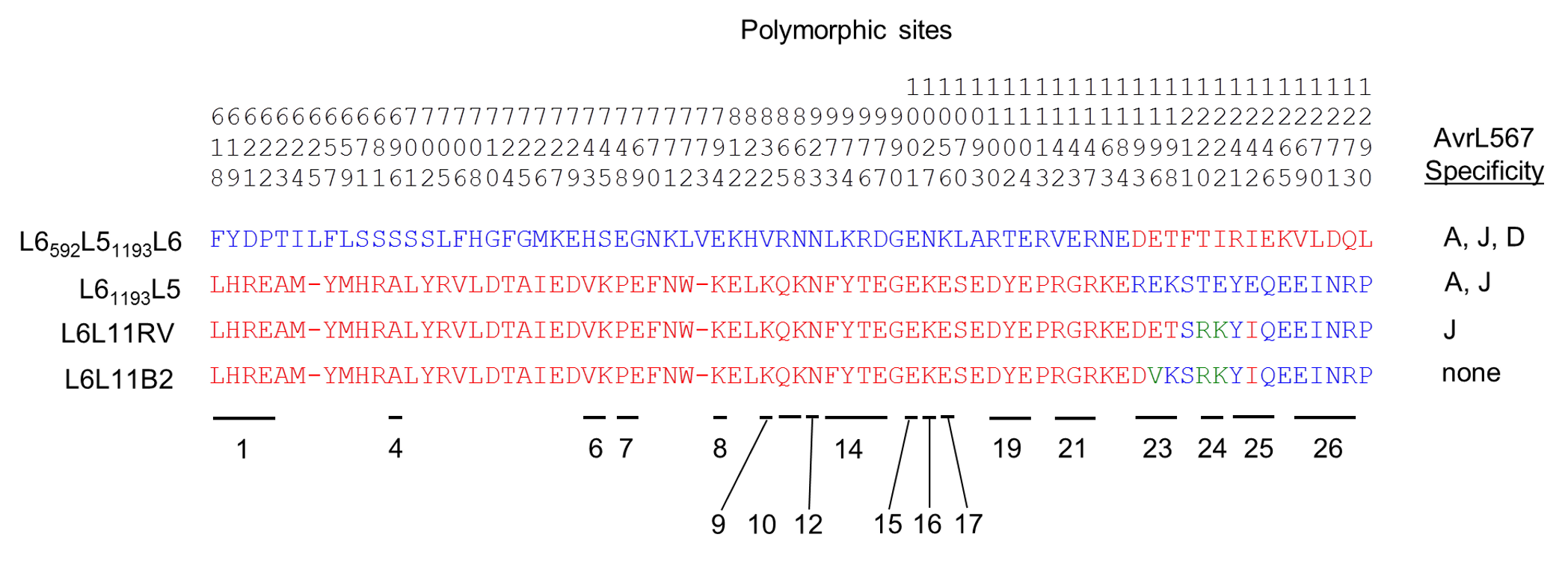

Supplement: Figure S7 — Polymorphic amino acid residues found in the LRR domain of chimeric L5–L6 and L6–L11 [56] resistance proteins. Residues from L5 are shaded blue, those from L6 are shaded red, and those from L11 are shaded green. LRR subunits with polymorphic residues in the β-strand/β-strand structure (xxLxLxx motif) are marked with black bars and are numbered below. All residues are listed below the corresponding L5 residues. (TIF) [file ppat.1003004.s007.tif]

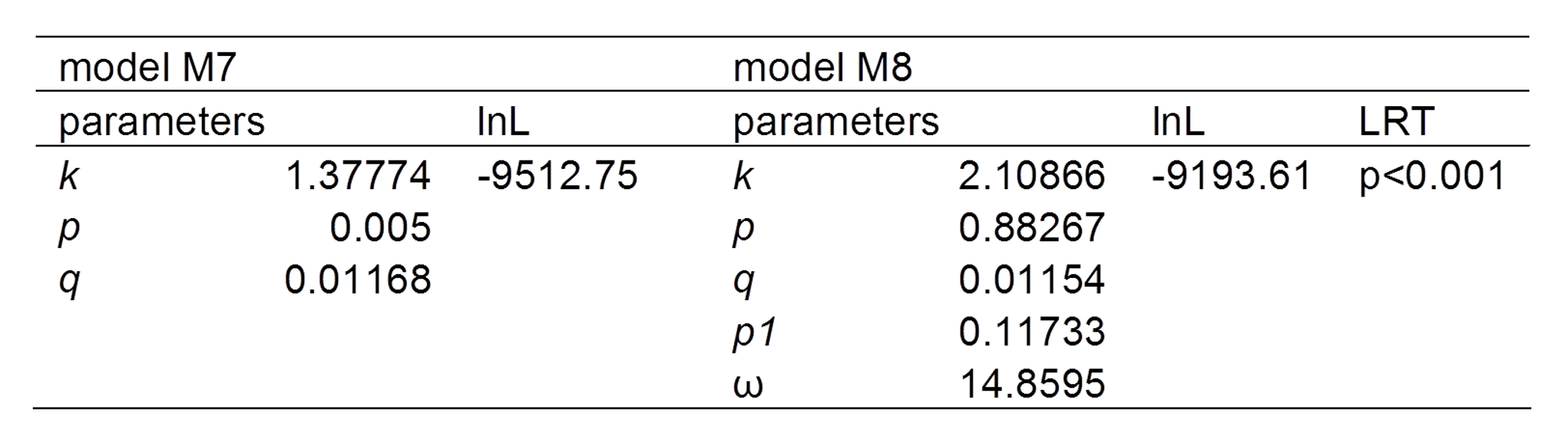

Supplement: Table S1 — Log-likelihoods (lnL), Log-likelihoods ratio test (LRT), and estimates of the model parameters (κ: transition/transversion rate ratio; parameters of the beta distribution: p and q ; p1: the fraction of sites estimated to fall within the class of sites ω>1, and the mean value (ω) for that class) for the models M7 and M8 in CODEML. (TIF) [file ppat.1003004.s008.tif]
