# Supplementary material for: Intramolecular Interaction Influences Binding of the Flax L5 and L6 Resistance Proteins to their AvrL567 Ligands
Source: PLoS Pathog. 2012 Nov 29;8(11):e1003004. doi: 10.1371/journal.ppat.1003004 (PMC3510248; doi:10.1371/journal.ppat.1003004)
Supplement: Text S1 — Supplementary experimental procedures. (DOC) [file ppat.1003004.s009.doc]

**Text S1 Supplementary Experimental Procedures**

**DNA manipulation for the construction of recombinant genes**

Unless otherwise noted, all constructs are contained in pGADT7.

Construct L5_185_L6 was generated using PCR-based fusion of overlapping sequences. L5 was used as a template with the primers 5’ AD (CTATTCGATGATGAAGATACCCCACCAAACC) and 3’ 192 (agcatctttccagttttgtatggt), and L6 was used as a template with the primers 5’ 185 (accatacaaaactggaaagatgct) and 3’ L6-P2. PCR products were isolated using agarose gel electrophoresis, and a mixture containing 100 ng of each product was utilized as a template for a PCR reaction using the primers 5’ AD and 3’ L6-P2. This product was cloned into pBSK using blunt-end ligation, and the *Eco*RI fragment was cut from this vector and ligated in to *Eco*RI*-*digested L6. Construct L5_226_L6 was constructed similarly using the primers 5’ AD and 3’ 233 (ttctaaaatgagattttccttgct), and 5’ 226 (agcaaggaaaatctcattttagaa) and 3’ L6-P2; as was L5_556_L6 using the primers 5’AD and 3’ TIR-NBS (ATGCGCGAGCTCAGGCCTCTTTTTGTTCAACAAGAGAT), and 5’ LRR (tatgggtagagaaattgtga) and 3’ L6-P2.

The constructs L5_414_L6 and L5_431_L6 were generated as follows: without changing protein sequences, unqiue *Sal*I sites at positions 352, and 431 were introduced into L6 using by PCR using the primers 5' L6-353 (ATGCGCGTCGACgagaagtttaaatttgaagat), 5' L6-431 (ATGCGCGTCGACactacagcaggacttccattg), and 3’ L6-P2, using L6 as a template; and into L5 using 5’ AD and 3' L5-352 (ATGCGCGAGCTCGTCGACatcatcgagaacgacaagaat), and 3' L5-430-RS (ATGCGCGAGCTCGTCGACgacatcatttgctagagtctc) using L5 as a template. These PCR fragments also contained an introduced *SacI* site at their 3’ terminus. PCR fragments were cloned into pBSK using blunt-end ligation. 5’ L5 fragments were cut from pBSK and ligated into pGADT7 using *Eco*RI and *Sac*I restriction sites. 3’ L6 fragments were cut from pBSK using *Sal*I and *Sac*I restriction sites and ligated into the pGADT7 vectors containing 5’ L5 fragments. Chimeric L5-L6 fragments were then cut from these pGADT7 vectors using *Eco*RI and ligated into pGADT7-L6 that had been digested with *Eco*RI*.*

L5_592_L6 and L6_592_L5 were constructed as follows: using L5 and L6 as templates, a unique *Avr*II site was introduced at position 593 was by PCR into L5 and L6. A fragment containing the TIR-NB-ARC region with added 3’ *Avr*II and *Sac*I sites was amplified using the primers 5’ AD and 3' L5-TIR-NBS-RS (ATGCGCGAGCTCCCTAGGcggcaccatgctaattgcttt); and a fragment containing the LRR domain with an added 5’ *Avr*II site was amplified using 5' L5-LRR-RS (ATGCGCCCTAGGgtgtcatgggataataatgtc) and 3’ AD (agatggtgcacgatgcacag). These fragments were blunt-end ligated into pBSK. Fragments containing the TIR-NB-ARC region of either L5 or L6 were cut from pBSK using *EcoRI* and *SacI* and were ligated into pGADT7. Fragments containing either the L5 or L6 LRR domain were cut from pBSK using *Avr*II and *Sac*I and were ligated into the pGADT7 vectors containing the TIR-NB-ARC region, cut with *Avr*II and *Sac*I*.*

The constructs L6_414_L5, L6_431_L5 and L6_493_L5 were constructed in a manner similar to L5_414_L6, L5_431_L6 and L5_493_L6 except the initial L5 and L6 templates were swapped, and 3' L5-TIR-NBS-RS was used instead of of 3’ L6-P2. This resulted in chimeric L6-L5 TIR-NBS-ARC constructs with a 3’ *Avr*II site. *Eco*RI and *Avr*II*-*digested fragments were then ligated into *Eco*RI and *Avr*II digested pGADT7 L6_592_L5. L5_185_L6_592_L5 was constructed using this method, with L5_185_L6 being the initial template.

L6_414_L5_556_L6 was generated in a manner similar to L6_414_L5, except that L5_556_L6 was used instead of L5 as an initial template, and 3’ L6-P2 was used instead of 3' L5-TIR-NBS-RS. The resulting product was cloned into pBSK using blunt end ligation, and an *Eco*RI fragment was cut from this vector and ligated into pGADT7 cut with *Eco*RI.

L5_793_L6, L5_972_L6, L5_1125_L6, L5_1193_L6; L6_793_L5, L6_972_L5, L6_1125_L5, L6_1193_L5; L6_592_L5_793_L6, L6_592_L5_972_L6, L6_592_L5_1125_L6, and L6_592_L5_1193_L6 were also constructed using PCR-based fusion of overlapping sequences. Using either L5_592_L6 or L6_592_L5 as a template, fragments of the 5’ end of the LRR domain were amplified using 5’ 493 (gatatagcttgcttcttcatcgga) and 3' 781 (aactacctctctcaaatttgtccc), 3' 971 (ccgcaacttgcaggtagaagaaca), 3' 1124 (tcctccaatgtctagctccttcag), or 3' 1192 (caactcttctagggatccaagcac). Using L5 or L6 as a template, fragments of the 3’ end of the LRR domain were amplified using 3’ AD and 5' 774 (gggacaaatttgagagaggtagtt) 5' 964 (tgttcttctacctgcaagttgcgg) 5' 1117 (ctgaaggagctagacattggagga) or 5' 1185 (gtgcttggatccctagaagagttg). Fragments were then mixed, and amplified using 5’ 493 and 3’ AD. The resulting fragments were cloned into pBSK using blunt end ligation, and chimeric LRR domains were cut out of this vector using *Avr*II and *Sac*I. These fragments where then ligated into either L5_592_L6 or L6_592_L5 digested with *Avr*II and *Sac*I*.*

L6_493_L5_793_L6, L6_493_L5_972_L6, L6_493_L5_1125_L6, and L6_493_L5_1193_L6 were constructed by cutting the chimeric LRR domains from L6_592_L5_793_L6, L6_592_L5_972_L6, L6_592_L5_1125_L6, and L6_592_L5_1193_L6 using *Avr*II and *Sac*I. These fragments where then ligated into L6_493_L5 digested with *Avr*II and *Sac*I*.*

L5_556_L6_793_L5, L5_556_L6_972_L5, L5_556_L6_1125_L5, and L5_556_L6_1193_L5 were constructed by ligating an *Eco*RI fragment from L5_556_L6 into *Eco*RI*-*digested pGADT7 L6_793_L5, L6_972_L5, L6_1125_L5, or L6_1193_L5. Similarly, L5_226_L6_793_L5, L5_226_L6_972_L5, L5_226_L6_1125_L5, L5_226_L6_1193_L5; L5_226_L6_592_L5_793_L6, L5_226_L6_592_L5_973_L6, L5_226_L6_592_L5_1125_L6 and L5_226_L6_592_L5_1193_L6 were constructed by ligating an *Eco*RI and *Avr*II fragment from cut L5_226_L6 into *Eco*RI- and *Avr*II*-*digested pGADT7 L6_793_L5, L6_972_L5, L6_1125_L5, L6_1193_L5, L6_793_L5, L6_972_L5, L6_1125_L5, or L6_1193_L5.

L6_185_L5_556_L6, L6_226_L5_556_L6, L6_414_L5_556_L6, and L6_493_L5_556_L6 were constructed using PCR-based fusion of overlapping sequences, using L6 and L5_556_L6 as templates, and the primers 5’ AD, 5’ 185, 5’ 226, 5’ 414 (ttcaaaaagaatacgcctccatcg), 5’ 493 (gatatagcttgcttcttcatcgga), 3’ L6-P2, 3’ 192, 3’ 233, 3’ 421 (cgatggaggcgtattctttttgaa) and 3’ 500 (tccgatgaagaagcaagctatatc). Products were mixed and used as a template for a PCR reaction using the primers 5’ AD and 3’ L6-P2. This product was cloned into pBSK using blunt-end ligation, and an *Eco*RI fragment was cut from this vector and ligated in to *Eco*RI*-*digested L6.

L6_185_L5_556_L6_592_L5_793_L6 and L6_185_L5_556_L6_592_L5_1193_L6 were constructed as follows: a unique *Avr*II site was introduced at position 593 by PCR into L6_185_L5_556_L6. A fragment containing the TIR-NB-ARC region with added 3’ *Avr*II and *Sac*I sites was amplified using the primers 5’ AD and 3' L6-TIR-NBS-RS; these were blunt-end ligated into pBSK, and a *Eco*RI */ Sac*I fragment containing the TIR-NBS region was cloned into pGADT7 cut with *Eco*RI */ Sac*I, generating pGADT7 L6_185_L5_556_L6_592_*.* Fragments containing chimeric LRR domains were cut out of L6_592_L5_793_L6 and L6_592_L5_1193_L6 pGADT7 using *Avr*II and *Sac*I. These fragments where then ligated into either pGADT7 L6_185_L5_556_L6_592_ digested with *Av*rII and *Sac*I*.*

L5 and L6_493_L5_1193_L6 were modified to include the 5’-UTR of L6 constructed using PCR-based fusion of overlapping sequences, using L5, L6 and L6_493_L5_1193_L6 as templates, and the primers 5' L6-UTR (ATGCGCGAATTCaccggatcccccgagctcaga), 5' 27 (aattccaaagactcaatcgtcaac) and 3' 34 (gttgacgattgagtctttggaatt). Products were mixed and used as a template for a PCR reaction using the primers 5' L6-UTR and 3' 34. These products were cloned into pBSK using blunt-end ligation, and an *Eco*RI fragment was cut from this vector and ligated in to *Eco*RI*-*digested pTNotTReg.
